# MCT4-dependent lactate transport: a novel mechanism for cardiac energy metabolism injury and inflammation in type 2 diabetes mellitus

**DOI:** 10.1186/s12933-024-02178-2

**Published:** 2024-03-14

**Authors:** Xiu Mei Ma, Kang Geng, Peng Wang, Zongzhe Jiang, Betty Yuen-Kwan Law, Yong Xu

**Affiliations:** 1 Faculty of Chinese Medicine, Macau University of Science and Technology, Avenida Wai Long, Taipa, 999078 Macau People’s Republic of China; 2 State Key Laboratory of Quality Research in Chinese Medicine, Macau University of Science and Technology, Avenida Wai Long, Taipa, 999078 Macau People’s Republic of China; 3 Department of Endocrinology and Metabolism, The Affiliated Hospital of Southwest Medical University, Luzhou, 646000 Sichuan People’s Republic of China; 4 Cardiovascular and Metabolic Diseases Key Laboratory of Luzhou, Luzhou, 646000 Sichuan People’s Republic of China; 5 Sichuan Clinical Research Center for Nephropathy, Luzhou, 646000 Sichuan People’s Republic of China

**Keywords:** MCT4, Lactate, Lipotoxicity, Inflammation, Diabetic cardiomyopathy

## Abstract

Diabetic cardiomyopathy (DCM) is a major contributor to mortality in diabetic patients, characterized by a multifaceted pathogenesis and limited therapeutic options. While lactate, a byproduct of glycolysis, is known to be significantly elevated in type 2 diabetes, its specific role in DCM remains uncertain. This study reveals an abnormal upregulation of monocarboxylate transporter 4 (MCT4) on the plasma membrane of cardiomyocytes in type 2 diabetes, leading to excessive lactate efflux from these cells. The disruption in lactate transport homeostasis perturbs the intracellular lactate-pyruvate balance in cardiomyocytes, resulting in oxidative stress and inflammatory responses that exacerbate myocardial damage. Additionally, our findings suggest increased lactate efflux augments histone H4K12 lactylation in macrophages, facilitating inflammatory infiltration within the microenvironment. In vivo experiments have demonstrated that inhibiting MCT4 effectively alleviates myocardial oxidative stress and pathological damage, reduces inflammatory macrophage infiltration, and enhances cardiac function in type 2 diabetic mice. Furthermore, a clinical prediction model has been established, demonstrating a notable association between peripheral blood lactate levels and diastolic dysfunction in individuals with type 2 diabetes. This underscores the potential of lactate as a prognostic biomarker for DCM. Ultimately, our findings highlight the pivotal involvement of MCT4 in the dysregulation of cardiac energy metabolism and macrophage-mediated inflammation in type 2 diabetes. These insights offer novel perspectives on the pathogenesis of DCM and pave the way for the development of targeted therapeutic strategies against this debilitating condition.

**Supplementary Information:**

The online version contains supplementary material available at 10.1186/s12933-024-02178-2.

## Introduction

Cardiovascular disease is the primary contributor to the worldwide burden of disease. Within this category, diabetic cardiomyopathy (DCM) emerges as a significant complication encountered by individuals with diabetes, particularly prevalent in those with type 2 diabetes mellitus (T2DM) [1]. Given the rising incidence of diabetes, DCM is garnering increasing attention. DCM can result in diastolic dysfunction, compromising the heart’s efficacy in pumping blood. In severe cases, it can even lead to heart failure and fatality [2]. Characterized by its insidious onset, rapid progression, poor prognosis, and limited treatment options, investigating the pathogenic mechanisms and viable therapeutic targets of DCM carries profound clinical and societal significance.

The pathogenesis of DCM encompasses a multitude of intricate biological mechanisms, encompassing aberrant energy metabolism and subsequent oxidative stress, endoplasmic reticulum stress, inflammation, apoptosis, and fibrosis [3]. Prior investigations have predominantly concentrated on cardiac fatty acid, glucose, and ketone body metabolism, with lactic acid previously regarded as a byproduct of anaerobic metabolism and receiving less scrutiny. In recent studies, the significance of lactic acid in maintaining metabolic homeostasis in aerobic conditions has been established, highlighting its three primary biological roles: serving as the primary fuel for the tricarboxylic acid cycle (TCA cycle), acting as a precursor for gluconeogenesis, and functioning as a signaling molecule. Lactic acid exhibits cellular mobility, facilitating the distribution of energy substrates and the transmission of cellular signals, thereby exerting a crucial influence on the development of numerous diseases, including cancer and obesity [4, 5].

Recently, there has been a conspicuous escalation in academic curiosity surrounding the functional importance of lactic acid in cardiac tissue. At rest, the heart predominantly utilizes free fatty acids as its energy source, with lactic acid playing a minor role. However, during physical exertion, lactic acid’s contribution to energy production undergoes a notable amplification [6]. Additionally, metabolites derived from lactic acid serve as valuable oxidizable substrates, effectively mitigating cellular glucolipotoxicity [7]. Macrophages, as the inherent immune cells of the heart, hold substantial significance in diverse pathological mechanisms linked to T2DM, such as myocardial injury, inflammation, and fibrosis [8–10]. Emerging research reveals that lactic acid can induce histone lactylation in macrophages across multiple tissues, including the heart. This process subsequently mediates the reprogramming of macrophage phenotypes and participates in a wide range of disease processes [11–13].

Based on the preceding discoveries, we put forward a potential therapeutic approach: by elucidating the precise role of lactic acid in cardiac tissue and identifying targets involved in regulating lactic acid transport, our objective is to ensure optimal distribution of cardiac lactic acid, thereby mitigating diabetic-induced myocardial injury and inflammation. To delve deeper into this subject, we established a mouse model of type 2 diabetic cardiomyopathy and employed cardiac transcriptome sequencing alongside bioinformatics analysis techniques. Our findings uncovered an upregulation of key differential genes encoding MCT4, a crucial lactate transporter. In vivo and in vitro experiments delved into the significance and underlying mechanisms of MCT4-dependent lactate transport in type 2 diabetic heart injury and inflammation. Furthermore, we established the beneficial effects of MCT4 inhibition in alleviating diabetic cardiomyopathy. These insights not only bolster the theoretical foundation for developing novel therapeutic avenues targeting diabetic cardiomyopathy but also pave the way for future clinical investigations.

## Methods

### Regents and antibodies

In this study, we used the following antibodies: anti-MCT4 (Cat No. 22787-1-AP, Proteintech, USA; abs124388, absin, China), anti-MCT1 (abs 120479, absin, China), anti-α-actinin (sc-17829, Santa Cruz, USA), anti-BNP (13299-1-AP, Proteintech, USA), anti-NPPA (27426-1-AP, Proteintech, USA), anti-Pan Kla (PTM-1401RM, PTM, China), anti-H3K18La (PTM-1406RM, PTM, China), anti-H4K12La (PTM-1411RM, PTM, China), anti-CD68 (sc-20060, Santa Cruz, USA), anti-iNOS (#13120, CST, USA), anti-Arg-1 (#93668, CST, USA), anti-HIF-1α (#14179, CST, USA), anti-IL-1β (#12242, CST, USA), anti-Mitofilin (ab110329, Abcam, USA), and anti-dsDNA (HYB331-01, Santa Cruz, USA). For immunoblotting experiments, horseradish peroxidase (HRP)-labeled goat anti-mouse IgG (A0216, Beyotime, China) and goat anti-rabbit IgG (A0208, Beyotime, China) were used as secondary antibodies. For immunofluorescence experiments, Goat anti-mouse IgG and Goat anti-rabbit IgG (Alexa Fluor® Plus 48, Alexa Fluor® Plus 488, 555647) purchased from Thermo Fisher Scientific were used as secondary antibodies. Palmitic acid and lactic acid were purchased from Sigma (USA), and MCT4 inhibitor VB124 was obtained from Selleck (USA).

### Human study

This study received approval from the Ethics Review Committee of the Affiliated Hospital of Southwest Medical University (KY2023142). It was conducted in adherence to the principles outlined in the Declaration of Helsinki II. The study encompassed a total of 1097 patients diagnosed with T2DM at the Department of Endocrinology, Affiliated Hospital of Southwest Medical University, during the period spanning from November 2018 to August 2022. The exclusion criteria encompassed individuals with type 1 diabetes and other types of diabetes, those experiencing severe diabetic complications such as severe infections, hyperthyroidism, hypothyroidism, moderate to severe anemia, patients with other types of organic heart diseases such as congenital heart disease, coronary atherosclerotic heart disease, rheumatic heart disease, and those diagnosed with malignant tumors.

### Animal study

Male Lepr^db^ mice and their control group, m Lepr^db^ mice (6 weeks old), were purchased from Gempharmatech Co., Ltd., in China. To ensure a good breeding environment, all mice were raised under specific pathogen-free (SPF) conditions, with strict control of environmental humidity within the range of 50 ± 5% and temperature maintained between 20 and 22 °C. To induce diabetic cardiomyopathy, Lepr^db^ mice received a high-fat diet (provided by HFKbio, China) for 12 weeks, with fat content accounting for 60% of total energy. Meanwhile, the control group, m Lepr^db^ mice, received a standard diet. Throughout the experiment, we regularly measured the mice’s body weight and fasting blood glucose every 2 weeks and recorded relevant data in detail. Starting from the 9th week of the experiment, Lepr^db^ mice in the treatment group were administered intraperitoneal injections of the MCT4 inhibitor VB124 at a dose of 10 mg/kg/daily for 4 weeks of continuous treatment. Lepr^db^ mice in the model group received intraperitoneal injections of an equivalent volume of solvent. All animal experimental procedures involved in this study were conducted in accordance with relevant regulations of the National Institutes of Health (NIH) and have been approved by Southwest Medical University (approval number: 20220218-021).

### Echocardiography

Select the mice to be examined and ensure they acclimate to the experimental environment. Anesthetize the mice using 3% isoflurane and place them on a heating pad to maintain a stable body temperature. Connect the 30 MHz probe to the Vevo 3100 ultrasound system (VisualSonics, Canada) and perform the necessary warm-up and calibration steps. Subsequently, anesthetize the mice again and use a depilatory agent to remove hair from their chest area. Place the mice on their backs on a heated stage set to 40 °C, apply pure water coupling medium to their limbs to enhance electrical conductivity, and secure them in the appropriate position using tape. Adjust the operating table’s height so that the mouse’s head is slightly higher than the tail to ensure that the apex and base of the heart are at the same level. Apply conductive gel to the mouse’s chest and insert the probe into the gel for measurement. Collect and record relevant cardiac function parameters, including ejection fraction (EF), fractional shortening (FS), and peak E/A ratio.

### Isolation and culture of primary cardiomyocytes

The Mouse Primary Cardiomyocyte Isolation Kit (88281) was purchased from Thermo Fisher Scientific, and the specific operation method is referred to in the enclosed instructions. Briefly, we first cut freshly dissected neonatal mouse hearts into small pieces of 1–3 mm^3^ and washed them with HBSS. Next, we added recombinant cardiomyocyte isolation enzyme 1 (containing papain) and isolation enzyme 2 (containing collagenase) to ensure that the tissue was fully enzymatically digested into individual cells. After completion of enzymatic digestion, we performed cell yield and viability determinations. Finally, we cultured the cells in a medium containing DMEM, 10% fetal bovine serum (FBS), 1% penicillin/streptomycin (P/S), and 1‰ cardiomyocyte growth supplement and incubated them in an incubator at a temperature of 37 °C and a CO_2_ concentration of 5%.

### Cell culture

The original clonal cell line derived from embryonic BD1X rat heart tissue (H9C2) and mouse monocytic macrophage leukemia cells (RAW264.7) were both purchased from the American Type Culture Collection (ATCC) cell bank. H9C2 cells were cultured in high-glucose DMEM medium (Gibco, USA) containing 10% fetal bovine serum (FBS), while RAW264.7 cells were cultured in RPMI medium (Gibco, USA) containing 5.6 mmol/L glucose and 10% FBS. Both cell types were incubated in an incubator at 37 °C and a CO_2_ concentration of 5%. We utilized the transwell system (Corning, USA) for co-culture experiments. Specifically, RAW264.7 cells were added to the upper chamber of the transwell and cultured in RPMI medium without FBS. The co-culture experiments employed transwell inserts with a pore size of 0.4 μm, while macrophage migration assays used transwell inserts with a pore size of 8 μm.

### Knockdown of MCT4

Using Lipofectamine 3000 (Invitrogen, USA), small interfering RNA (siRNA) targeting rat MCT4 (Sense 5′ → 3′: CCUGCUAGACCUGAGUGUCUUdTdT, antisense 5′ → 3′: AAGACACUCAGGUCUAGCAGGdTdT, Hippobiotec, China) was transfected into H9C2 cells. After 72 h of incubation, experiments were conducted, including crystal violet staining, immunofluorescence staining, and RNA extraction.

### Metabolism assays

According to the protocol provided by the manufacturer, we determined the concentrations of lactic acid and pyruvic acid in the medium or cells using either the l-Lactic Acid Assay Kit or the Pyruvic Acid Assay Kit (Solarbio, China).

### Cellular ROS and ATP assay

According to the manufacturer’s instructions, we utilized the DCFH-DA Cellular ROS Detection Kit (Beyotime, China), MitoSOX Red Mitochondrial Superoxide Indicator (YEASEN, China), and ATP Detection Kit (Beyotime, China) to separately measure the levels of reactive oxygen species (ROS) and ATP within the cells.

### Mitochondrial membrane potential, Λψm

Λψm was measured using mitochondrial-specific fluorescent dye JC-1 staining (Solarbio, China). The procedure was as follows: cells were seeded in 24-well plates and treated accordingly when the confluence reached 80%. The treatment time was determined based on experimental requirements. Subsequently, the cells were washed twice with PBS and then incubated with a medium containing JC-1 for 30 min. The fluorescence intensity of JC-1 aggregates and JC-1 monomers was detected by excitation/emission wavelengths of 585 nm/590 nm and 515 nm/529 nm, respectively. The fluorescence intensity ratio between JC-1 aggregates and JC-1 monomers was used to indicate Λψm.

### Flow cytometry

After 24-h treatment with PA and/or lactic acid, the concentration of macrophages must be adjusted to 1 × 10^7^ cells/ml. From both the sample and control tubes, 100 μl of cell suspension is transferred to a new tube, followed by the addition of 5 μl of Anti-Mouse CD86 antibody (E-AB-F0994E, Elabscience, China). The mixture is then incubated for 60 min at 4 °C in the dark. Subsequently, FIX & PERM Medium A and B, as well as Anti-Mouse CD206 antibody (E-AB-F1135D, Elabscience, China), are added sequentially with mixing and incubation steps in between. During this process, centrifugation and washing are required to remove excess reagents. Finally, the cells are resuspended in a flow cytometry staining buffer for detection using the CytoFLEX flow cytometer (Beckman Coulter, USA).

### Histological and immunofluorescence staining

The heart tissue was immersed in a 4% formaldehyde PBS solution for 48 h to ensure adequate fixation. Subsequently, a series of processing steps were carried out, including dehydration, paraffin embedding, and sectioning of the tissue into 5-µm-thick slices. These sections underwent dewaxing and hydration treatments, followed by hematoxylin and eosin staining (Beyotime, China) or immunofluorescence staining. For immunostaining, the sections were first immersed in PBS containing 10% BSA (pH 7.4) for 1 h to block non-specific binding sites. Primary antibody (dilution ratio 1:100) was then added to the sections and incubated overnight at 4 °C to ensure adequate binding of the antibody to the target antigen. On the second day, the slides were washed three times with PBST buffer to remove unbound primary antibodies. Subsequently, fluorophore-labeled secondary antibody was incubated with the sections in the dark for 1 h to ensure binding to the primary antibody. Finally, we used Gold Antifade Mountant containing DAPI (Abcam, USA) to counterstain the slides, enhancing the nuclear staining of the cells. Images were captured using an Olympus fluorescence upright microscope (for HE), and a Leica inverted confocal microscope (for immunofluorescence).

### Protein extraction and Western blotting

Previous study [14] has elaborated on the process of protein extraction from cells and subsequent immunoblotting. In this study, we employed the following steps for tissue extracts: firstly, fine dissection of mouse heart tissue; secondly, thorough homogenization of the tissue using radioimmunoprecipitation assay (RIPA) lysis buffer containing 1% protease inhibitors to ensure complete lysis; thirdly, centrifugation of the tissue suspension at 12,000 rpm/min for 15 min; finally, after centrifugation, we collected the supernatant and determined its protein concentration to ensure suitability for subsequent immunoblotting experiments.

### RNA isolation, RT-PCR, and RNA sequencing

Previous research [14] has provided detailed steps for RNA isolation, reverse transcription PCR (RT-PCR), and real-time quantitative PCR. For the implementation and in-depth analysis of RNA sequencing (RNA-seq), we chose Majorbio, located in Shanghai, China, to conduct the study. The experimental procedure involved collecting heart tissue samples from mice and immediately snap-freezing them in liquid nitrogen to preserve sample integrity. Efficient RNA extraction was then carried out using a Trizol reagent. To ensure the quality of the extracted RNA, precise quality assessment was performed using a 2100 Bioanalyzer (Agilent, Santa Clara, CA), and accurate quantification was done using an ND-2000 (Thermo Fisher Scientific). Subsequently, we carefully prepared the RNA-seq transcriptome library following the guidelines provided by the TruSeq RNA Sample Prep Kit from Illumina (San Diego, California). Finally, all libraries were sequenced on the advanced Illumina HiSeq Xten NovaSeq 6000 sequencer, employing a read length of 2 × 150 base pairs.

### RNA-seq analysis

During the bioinformatics analysis stage, we employed the TPM (Transcripts Per Million) method to calculate the expression levels of transcripts. Both data analysis and visualization were conducted using R software version 4.2.2. To gain deeper insights into the data, we performed Gene Ontology (GO) and Kyoto Encyclopedia of Genes and Genomes (KEGG) analyses using the cluster profile package. Additionally, we conducted gene set enrichment analysis (GSEA) using the clusterProfiler package and msigdbr package. Immune infiltration analysis was accomplished using the CIBERSORT tool. To gain a more comprehensive understanding of protein interactions, we carried out relevant analyses using the STRING database (https://string-db.org/) and visualized the results using Cytoscape version 3.7.2. Furthermore, we utilized the Cytohubba plugin to identify hub genes for a deeper investigation of core genes and their interaction networks.

### Statistical analysis

For continuous data, we typically use mean and standard deviation to describe normal distributions, while for skewed distributions, we prefer to characterize them using median (M) and interquartile range (P25–P75). On the other hand, categorical data often present the proportional relationship between categories in the form of percentages, which helps us understand the data distribution more intuitively. In this study, all statistical analyses were performed using R software version 4.2.2. Specifically, we employed a two-tailed t-test for comparisons between two groups and chose a one-way analysis of variance (ANOVA) for pairwise comparisons among three groups. All statistical tests were two-sided, and significance levels were indicated by symbols: * for P < 0.05, ** for P < 0.01, and *** for P < 0.001.

## Results

### Transcriptome analysis of type 2 diabetic cardiomyopathy model: discovering the role of the key gene *Slc16a3*

**Fig. 1:**
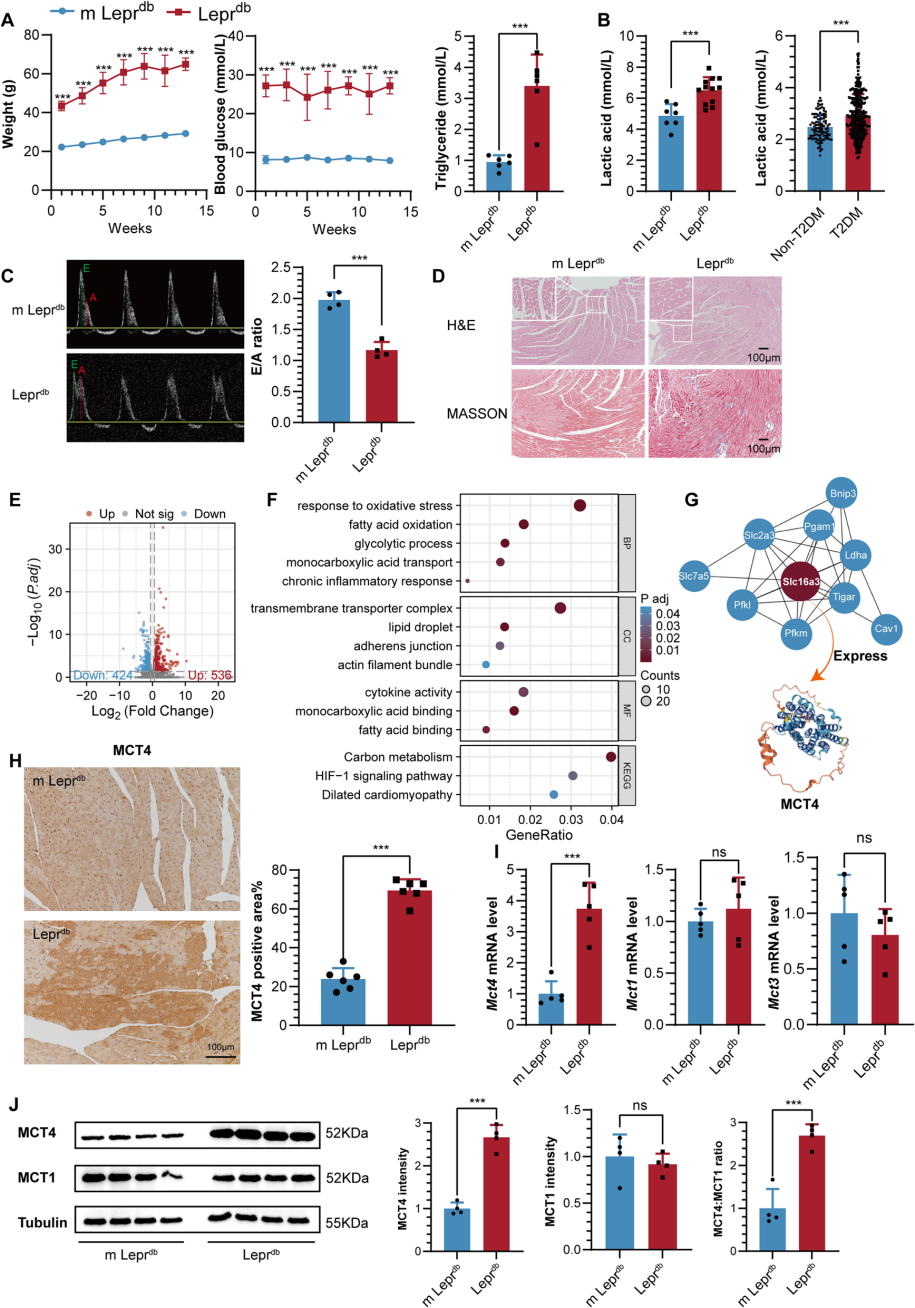
**A** Body weight, blood glucose and triglycerides in m Lepr^db^ and Lepr^db^ mice. **B** Blood lactic acid in Lepr^db^ vs. m Lepr^db^ mice and T2DM vs. non-T2DM. **C** Pulsed‐wave Doppler showing diastolic function: peak velocity of the E wave and A wave, E/A ratio in Lepr^db^ vs. m Lepr^db^ mice. **D** HE and Masson staining of the heart in Lepr^db^ vs. m Lepr^db^ mice. **E** Volcano plot showing differentially expressed genes (DEGs) of Lepr^db^ vs. m Lepr^db^ mice. **F** GO and KEGG analysis. **G** Protein–protein interaction (PPI) hub networks of Slc16a3. **H** MCT4 immunohistochemical staining of heart tissue in Lepr^db^ vs. m Lepr^db^ mice. **I** Cardiac *Mct1, 3, 4* mRNA expression in Lepr^db^ vs. m Lepr^db^ mice. **J** Western blot analysis and quantification of MCT4, MCT1, and MCT4:MCT1 ratio in Lepr^db^ vs. m Lepr^db^ mice

After a 12-week feeding period with a diet of 60% high fat, Lepr^db^ mice exhibited notable elevations in body weight, blood glucose, and triglyceride levels compared to control mice (m Lepr^db^) maintained on a standard diet. This precisely recapitulated the metabolic hallmarks of obesity, hyperglycemia, and hyperlipidemia observed in T2DM (Fig. 1A). Comprehensive investigations further unveiled that both Lepr^db^ and T2DM patients exhibited notably elevated blood lactate levels relative to the control group (Fig. 1B). Echocardiographic assessments revealed a reduced E/A ratio in Lepr^db^ mice, serving as a distinct indicator of diastolic dysfunction (Fig. 1C). Furthermore, pathological staining techniques highlighted increased myocardial thickness and broadened fibrotic areas in Lepr^db^ mice (Fig. 1D). Collectively, these findings corroborate the successful establishment of a mouse model for type 2 diabetic cardiomyopathy.

We conducted transcriptome sequencing analysis on heart tissue samples obtained from both Lepr^db^ and m Lepr^db^ mice. By applying a threshold of an absolute Log_2_FC value greater than 0.585 and an adjusted P-value less than 0.05, we identified a total of 536 upregulated genes and 424 downregulated genes (Fig. 1E). Subsequent GSEA analysis revealed that these differentially expressed genes (DEGs) were predominantly enriched in biological processes related to interferon signaling, innate immunity, viral myocarditis, and extracellular matrix (ECM) regulation (Additional file 1: Fig. S1A). These findings suggest a potential association between DCM, innate immune responses, and inflammatory processes.

Concurrently, immune infiltration analysis revealed significantly higher proportions of monocytes and macrophages in Lepr^db^ heart tissues than m Lepr^db^ (Additional file 1: Fig. S1B), indicating a crucial role for monocyte-macrophages in the pathogenesis of DCM. Through GO analysis, we observed that these DEGs were primarily enriched in biological pathways involving fatty acid binding/oxidation, glycolysis, and monocarboxylic acid binding/transport. These processes are closely associated with oxidative stress response, cytokine activity, and chronic inflammation. Additionally, the KEGG analysis highlighted the involvement of these DEGs in biological processes such as carbon metabolism, HIF-1 signaling pathway, and dilated cardiomyopathy (Fig. 1F). These findings suggest a pivotal role for interconnected biological processes involving energy metabolism, oxidative stress, and inflammation in the development of DCM. Furthermore, by leveraging protein–protein interaction analysis and the cytohub plugin, we identified a key gene, *Slc16a3*. This gene exhibits associations with multiple genes implicated in glycolysis metabolism, including *Ldha*, *Pfkl*, *Pfkp*, and *Tiger* (Additional file 1: Fig. S1C, Fig. 1G). According to annotations from the UniProt database (https://www.uniprot.org/), the protein product of the *Slc16a3* gene is MCT4, which possesses a pore structure (Fig. 1G) and facilitates the transport of monocarboxylic acids such as lactate.

Immunohistochemical experiments convincingly demonstrated a marked increase in MCT4 expression in Lepr^db^ heart tissues compared to m Lepr^db^ (Fig. 1H). This observation is consistent with our RT-qPCR analysis, which further substantiates the upregulated trend of *Mct4* in the Lepr^db^ (Fig. 1I). Notably, the RT-qPCR analysis revealed no significant variations in the expression of *Mct1* and *Mct3* between the two groups (Fig. 1I). Given that MCT4 and MCT1 are responsible for lactate efflux and influx, respectively, we further examined the expression of these two proteins in mouse heart tissue using Western blot and calculated their ratio. The results showed that in Lepr^db^ mice, the expression level of MCT4 and the ratio of MCT4 to MCT1 were significantly higher than those in the control group. In contrast, the expression level of MCT1 remained stable between the two groups (Fig. 1J). These discoveries strongly implicate the upregulated expression of MCT4 as a potential key factor in the development of DCM.

We postulate that this phenomenon might be intimately linked to the process of lactate transport across the plasma membrane. To investigate this hypothesis, we further isolated the plasma membrane from heart tissue and re-evaluated MCT4 expression. The results were consistent, revealing a significantly higher expression of MCT4 in the heart plasma membrane of Lepr^db^ mice compared to the control group (Additional file 1: Fig. S1D). These experimental observations collectively implicate a pivotal role of MCT4 located on the myocardial plasma membrane in regulating lactate transport and energy homeostasis, thereby hinting at its potential involvement in the pathophysiology of DCM.

### Mechanism of fatty acid-induced cardiomyocyte injury: MCT4-mediated imbalance of lactate-pyruvate axis

**Fig. 2:**
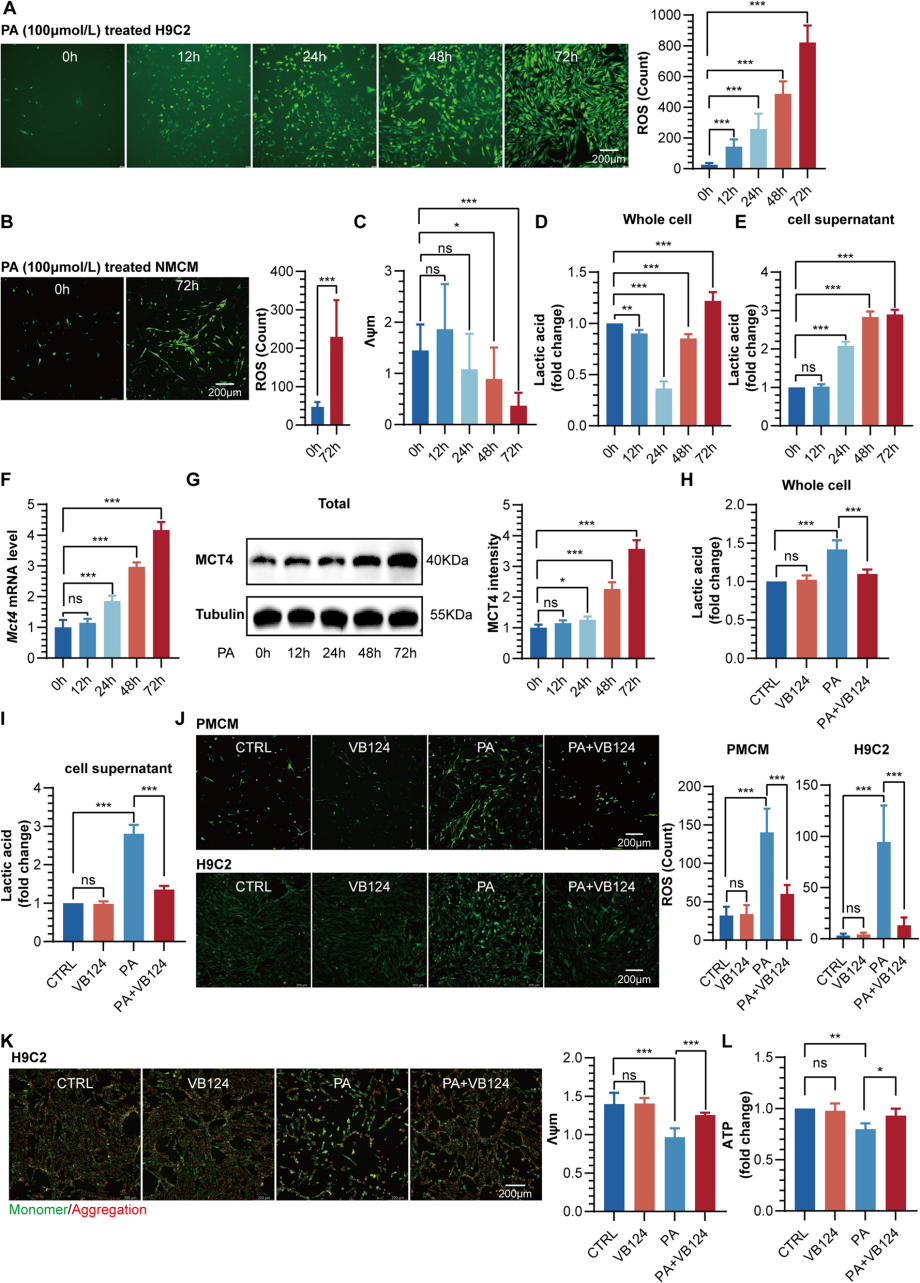
**A** Representative diagram and quantification of ROS using DCFH-DA staining in H9C2 cells treated with palmitic acid (PA). **B** Representative diagram and quantification of ROS in PMCM cells treated with PA. **C** Quantification of Λψm (mitochondrial membrane potential) in H9C2 cells treated with PA. **D** Fold change of lactic acid in H9C2 whole cell lysates after time-dependent PA stimulation. **E** Fold change of lactic acid in H9C2 cell supernatant after time-dependent PA stimulation. **F** *Mct4* mRNA expression in H9C2 cells treated with PA. **G** Western blot analysis and quantification of MCT4 in H9C2 cells treated with PA. **H** Fold change in whole-cell abundances of lactic acid in H9C2 treated with PA and/or VB124. **I** Fold change in cell supernatant abundances of lactic acid in H9C2 treated with PA and/or VB124. **J** Representative diagram and quantification of ROS in PMCM and H9C2 cells treated with PA and/or VB124. **K** Representative diagram and quantification of Λψm in H9C2 cells treated with PA and/or VB124. **L** Fold change of ATP content in H9C2 treated with PA and/or VB124

Fatty acid overload and lipotoxicity are critical factors triggering myocardial injury in T2DM [14, 15]. To mimic an in vitro environment of hyperlipidemia, we utilized PA to stimulate primary mouse cardiomyocytes (PMCM) or H9C2 cells. Our findings revealed a time-dependent elevation of reactive oxygen species (ROS) levels in H9C2 cells following PA stimulation (Fig. 2A). Additionally, we observed a significant decrease in Λψm after 24 h of PA stimulation (Additional file 1: Fig. S2A, Fig. 2C). Similarly, elevated ROS levels were also observed in PMCM (Fig. 2B), indicating that free fatty acids are the primary drivers of mitochondrial oxidative stress in cardiomyocytes.

Previous research has highlighted the crucial role of the lactate-pyruvate axis in regulating cardiac hypertrophy and heart failure [16]. To explore the effects of PA stimulation on the dynamic changes of this axis in cardiomyocytes, we designed and conducted a series of time-gradient experiments in H9C2 cells. The findings uncovered an intriguing phenomenon: upon PA stimulation, the intracellular lactate level initially decreased but subsequently increased gradually. Concurrently, the pyruvate level demonstrated an opposite trend, first increasing and then declining (Fig. 2D, Additional file 1: Fig. S2B). Moreover, we observed that PA caused a time-dependent increase in lactate concentration in the supernatant of cardiomyocytes (Fig. 2E), suggesting that PA may induce an imbalance in the lactate-pyruvate axis and cellular lactate efflux in cardiomyocytes.

MCT4, a vital transporter for lactate in cardiomyocytes, is typically expressed at low levels under healthy conditions, as reported in previous research [17]. Nevertheless, our experimental findings indicate that both *Mct4* mRNA and protein expression exhibited a time-dependent upregulation in H9C2 cells upon stimulation with PA (Fig. 2F, G). Additionally, we conducted a specific analysis of MCT4 expression in the plasma membrane fraction of H9C2 cells. The findings further confirm an enhanced expression of MCT4 on the plasma membrane in response to PA stimulation. (Additional file 1: Fig. S2C). These findings suggest that free fatty acids may disrupt the imbalance of the lactate-pyruvate axis in cardiomyocytes by inducing MCT4 upregulation. To validate this hypothesis further, we utilized VB124 to specifically inhibit MCT4 activity or employed siRNA to knock down MCT4 expression in H9C2 cells. The experimental results revealed that VB124 significantly reduced the PA-induced increase in lactate levels in whole cells and supernatants (Fig. 2H, I), decreased whole-cell pyruvate levels (Additional file 1: Fig. S2D), and increased mitochondrial pool pyruvate levels (Additional file 1: Fig. S2E). The effect of MCT4 knockdown was consistent with the treatment effect of VB124 (Additional file 1: Fig. S2F–I). Importantly, we also found that knockdown or inhibition of MCT4 could attenuate ROS production in cardiomyocytes induced by PA (Fig. 2J, Additional file 1: Fig. S2J), reverse the decrease of Λψm (Fig. 2K, Additional file 1: Fig. S2L), and enhance ATP production (Fig. 2L, Additional file 1: Fig. S2M). Furthermore, we detected mitochondrial superoxide using MitoSOX and similarly observed that inhibiting MCT4 reduced PA-induced mitochondrial superoxide production in both PMCM and H9C2 cardiomyocytes (Additional file 1: Fig. S2K). Taken together, these findings suggest that MCT4 upregulation may mediate the imbalance of the lactate-pyruvate axis induced by fatty acids, ultimately leading to mitochondrial oxidative stress damage in cardiomyocytes.

### Protective effects of inhibiting/knocking down MCT4 on free fatty acid-induced cardiomyocyte injury

**Fig. 3:**
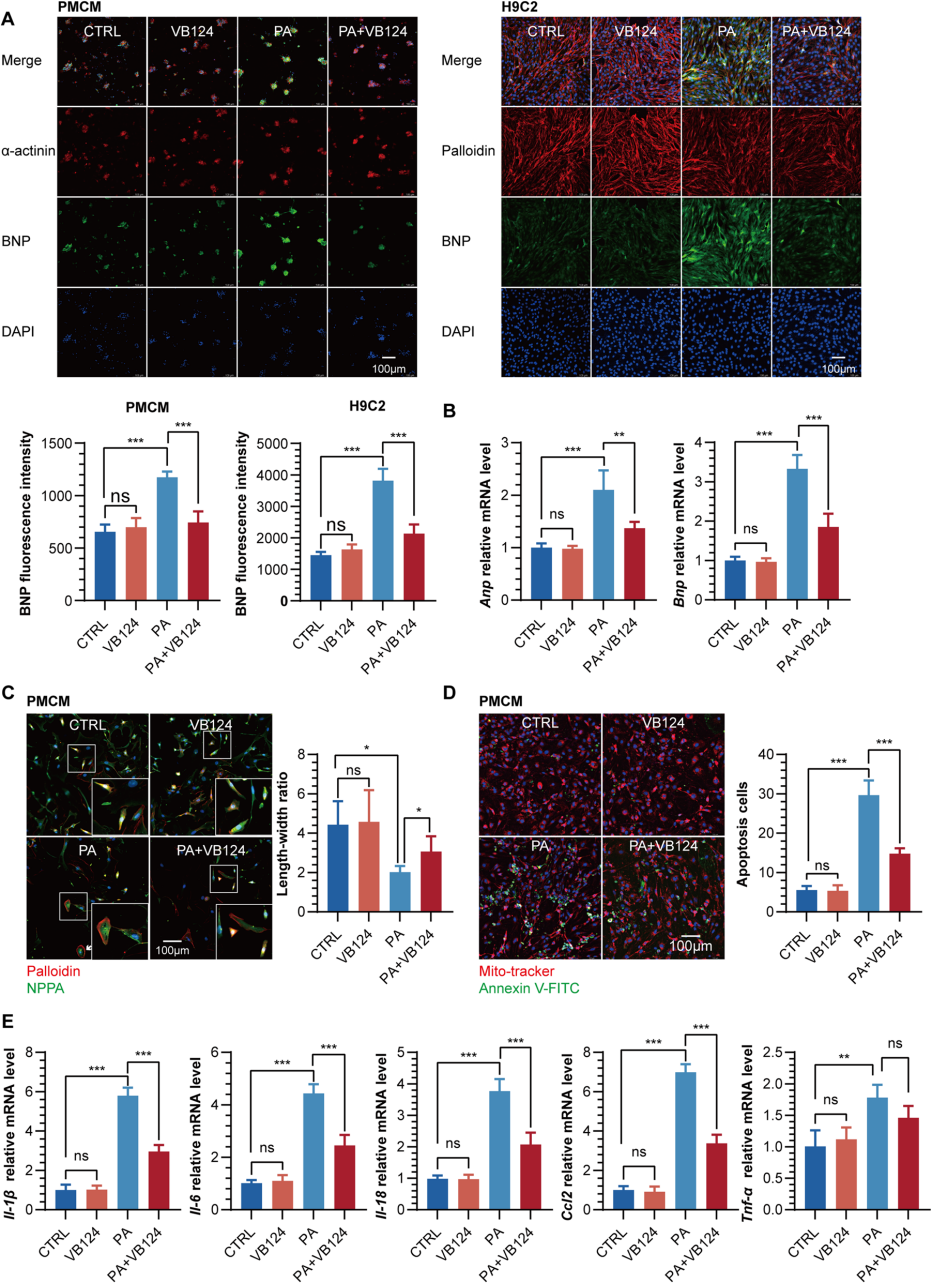
**A** Representative diagram and quantification of BNP in PMCM and H9C2 cells treated with PA and/or VB124. **B** *Anp* and *Bnp* mRNA expression in H9C2 cells treated with PA and/or VB124. **C** Representative diagram and quantification of length–width ratio in PMCM treated with PA and/or VB124. **D** Representative diagram and quantification of Annexin-V positive apoptosis cells in PMCM treated with PA and/or VB124. **E** mRNA expression of *Il-1β, Il-6, Il-18, Ccl2* and *Tnf-α* in H9C2 cells treated with PA and/or VB124

Given the intricate connections between oxidative stress injury, cardiac hypertrophy, inflammation, and apoptosis, we investigated the precise mechanisms of free fatty acid-induced damage in cardiomyocytes. We aimed to assess the potential protective effects of inhibiting or suppressing MCT4 against cardiomyocyte lipotoxicity. Utilizing immunofluorescence techniques, we quantified the expression levels of BNP in both PMCM and H9C2 cells. Notably, our results revealed a significant elevation of BNP expression upon PA exposure, which was effectively mitigated by VB124 treatment (Fig. 3A). Consistent with this observation, MCT4 knockdown in H9C2 cells exhibited a comparable effect to VB124 treatment (Additional file 1: Fig. S3A). Additionally, we examined the transcriptional profiles of *Anp* and *Bnp*, observing that both VB124 and MCT4 knockdown markedly reversed the PA-induced upregulation of *Anp/Bnp* mRNA (Fig. 3B, Additional file 1: Fig. S3B). By assessing the length-to-width ratio of cardiomyocytes, we further demonstrated that VB124 could substantially counteract the PA-induced reduction in this ratio in NMCM and H9C2 cells (Fig. 3C, Additional file 1: Fig. S3C), suggestive of an improvement in cardiac hypertrophy. Furthermore, we observed a marked increase in apoptosis rates in PA-exposed NMCM and H9C2 cells through apoptosis quantification, which was significantly attenuated by VB124 pretreatment (Fig. 3D, Additional file 1: Fig. S3D). Lastly, our analysis of inflammatory cytokine transcription levels revealed that pretreatment with either VB124 or MCT4 knockdown significantly reduced the mRNA levels of *Il-1β*, *Il-6*, *Il-18*, and *Ccl2* in H9C2 cells challenged with PA (Fig. 3E, Additional file 1: Fig. S3E). Collectively, our findings reveal the protective role of MCT4 inhibition or knockdown in reversing PA-induced cardiomyocyte hypertrophy and apoptosis while concurrently mitigating the transcription of inflammatory cytokines.

### Effects of MCT4-mediated lactate transport in cardiomyocytes on macrophage inflammatory response

**Fig. 4:**
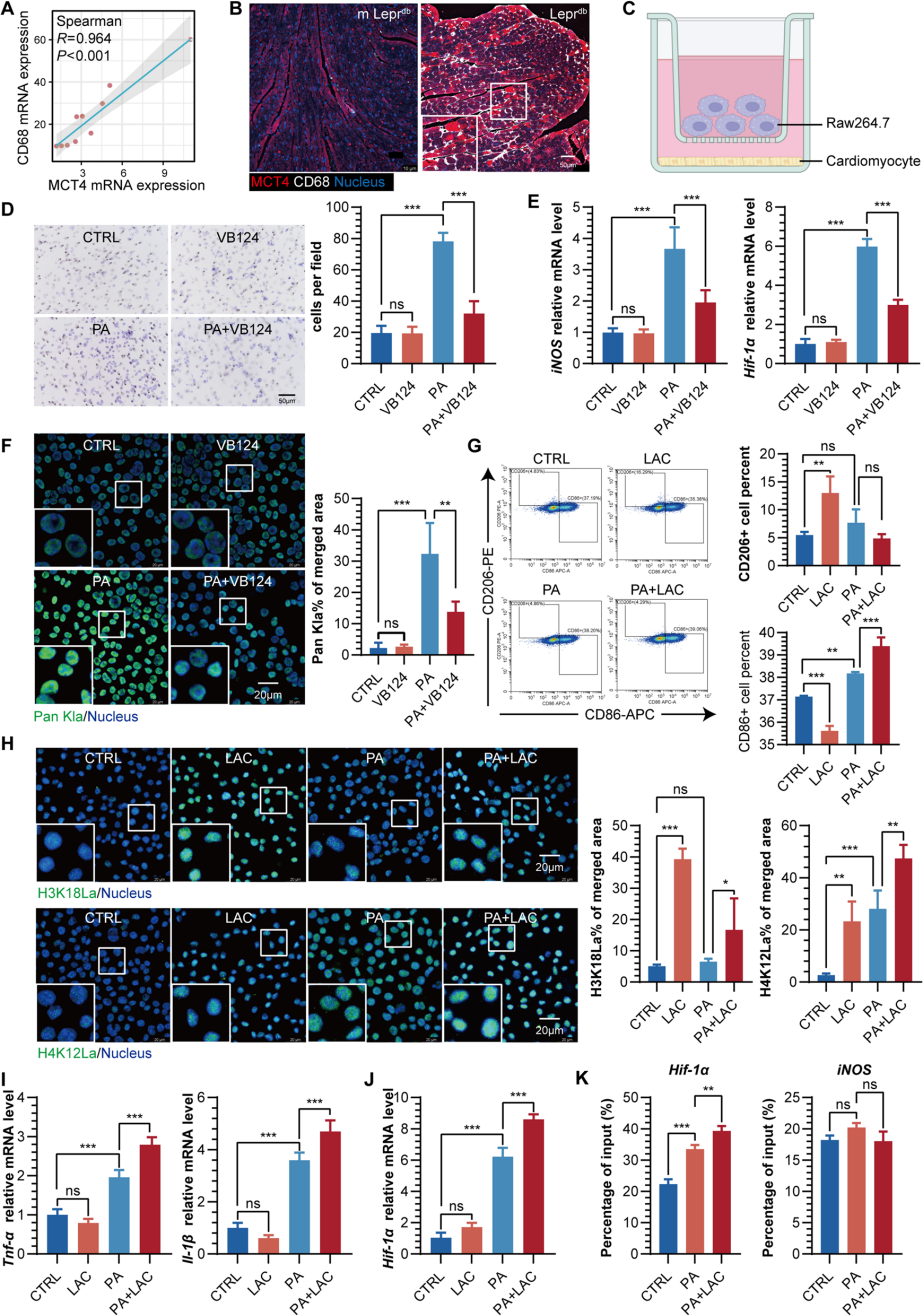
**A** Spearman correlation analysis between MCT4 and CD68 mRNA expression in the heart of mice. **B** Representative diagram of CD68 expression around the high expression of MCT4 in the heart of m Lepr^db^ and Lepr^db^ mice. **C** A diagram to depict the coculture experiment using transwell. RAW264.7 and H9C2 (pre-treated with PA and/or VB124) were cocultured for 24 h for **D**–**F**. **D** Representative diagram and quantification of migrated RAW264.7 cells stained with crystal violet. **E** mRNA expression of *iNOS* and *Hif-1α* in coculture RAW264.7. **F** Representative diagram and quantification of Pan Kla% in coculture RAW264.7. **G** Flow cytometry analysis of RAW264.7 macrophage subtypes treated with either PA, lactic acid (LAC), or a combination of both. **H** Representative diagram and quantification of H3K18La and H4K12La in RAW264.7 treated with PA and/or LAC. **I** mRNA expression of *Tnf-α* and* Il-1β* in RAW264.7 treated with PA and/or LAC. **J** *Hif-1α* mRNA expression in RAW264.7 treated with PA and/or LAC. **K** Chromatin IP (ChIP) quantitative real time PCR (ChIP-qRT-PCR) of gene targets for *HIF-1α* and *iNOS* at H4K12La in RAW264.7 treated with PA or PA + LAC

After conducting a correlation analysis of RNA-seq expression profiles, a positive association was observed between the macrophage marker CD68 and MCT4 (Fig. 4A). Immunofluorescence staining further enabled us to visualize the colocalization of MCT4 and CD68. Notably, we identified a prominent accumulation of CD68^+^ macrophages in regions exhibiting elevated MCT4 expression within Lepr^db^ heart tissue (Fig. 4B). These findings suggest that the upregulation of MCT4 in cardiomyocytes under T2DM conditions may play a role in promoting cardiac macrophage infiltration. To further elucidate how alterations in MCT4 within cardiomyocytes influence macrophages, we employed a Transwell co-culture method (Fig. 4C). Crystal violet staining indicated that when MCT4 in H9C2 cells was knocked down or inhibited, the migration of co-cultured macrophages induced by PA was significantly reduced (Fig. 4D, Additional file 1: Fig. S4A). Additionally, inhibiting MCT4 in NMCM cardiomyocytes also led to a substantial decrease in mRNA transcription levels of the inflammatory cytokines *Il-1β* and *Tnf-α* in PA-induced co-cultured macrophages (Additional file 1: Fig. S4B), as well as reduced mRNA transcription levels of the M1 macrophage polarization marker *iNOS* and hypoxia-inducible factor *Hif-1α* (Fig. 4E). Notably, the effects of MCT4 knockdown in H9C2 cells on macrophages were consistent with the impact of VB124 treatment (Additional file 1: Fig. S4C).

Given the pivotal role of MCT4 in lactate transport and the fact that lactate serves as a critical precursor in the process of histone lactylation, we hypothesize that knocking down or inhibiting the activity of MCT4 can effectively reduce the release of lactate from cardiomyocytes, subsequently decreasing the level of histone lactylation in adjacent infiltrating macrophages. To test this hypothesis, we employed immunofluorescence analysis and found that inhibiting MCT4 in NMCM cardiomyocytes significantly attenuates the enhanced histone lactylation observed in co-cultured macrophages induced by PA (Fig. 4F), further supporting our speculation. Based on these findings, we further hypothesize that changes in histone lactylation in macrophages may be closely related to their polarization states. To explore this hypothesis, we used RAW264.7 macrophages and treated them with lactic acid (LAC), PA, or a combination of both. Subsequent flow cytometry analysis revealed that lactic acid alone increases the proportion of CD206^+^ anti-inflammatory macrophages while decreasing the proportion of CD86^+^ pro-inflammatory macrophages. Conversely, PA alone induces an increase in the proportion of CD86^+^ pro-inflammatory macrophages. However, when lactic acid and PA are used in combination, we observe a further increase in the percentage of CD86^+^ pro-inflammatory macrophages (Fig. 4G).

It is well-established that arginase-1 (Arg-1) and inducible nitric oxide synthase (iNOS) are hallmark molecules of anti-inflammatory and pro-inflammatory macrophages, respectively. Therefore, monitoring the expression of these enzymes in macrophages is crucial for a deeper understanding of their polarization states and functions. Our immunofluorescence data align with the results obtained from flow cytometry, demonstrating that lactic acid alone upregulated the anti-inflammatory marker Arg-1 in macrophages, while PA enhanced the expression of the pro-inflammatory marker iNOS. Furthermore, the combined stimulation of lactic acid and PA led to further upregulation of iNOS expression (Additional file 1: Fig. S4D).

Simultaneously, we explored changes in lactic acid levels and histone lactylation in RAW264.7 macrophages under these stimulatory conditions. The results indicated that lactic acid alone significantly elevated intracellular lactic acid levels, accompanied by enhanced lactylation at the H3K18 and H4K12 sites. In contrast, while PA alone does not exert a notable influence on intracellular lactic acid levels, it specifically and substantially promotes lactylation at the H4K12 site. Notably, when lactic acid and PA are combined, not only does the intracellular lactic acid level increase, but the lactylation at the H4K12 site also exhibits a further enhancement trend (Fig. 4H, Additional file 1: Fig. S4E). These results suggest that exogenous lactic acid may enhance PA-induced pro-inflammatory polarization of macrophages by mediating H4K12 lactylation.

Further investigation revealed that PA could upregulate the transcription levels of various inflammatory factors in macrophages, such as *Tnf-α*, *Il-1β*, *Il-6*, and *Il-18* (Fig. 4I, Additional file 1: Fig. S4F). When combined with lactic acid, the transcription levels of *Tnf-α* and *Il-1β* were further amplified (Fig. 4I), confirming the role of lactic acid in enhancing PA-induced inflammatory responses in macrophages. Additionally, we observed a significant role for the hypoxia-inducible factor HIF-1α in this process, which is a crucial regulator of anti-inflammatory/pro-inflammatory polarization in macrophages. Our study found that under PA stimulation, the transcription level of *Hif-1α* increased, and when combined with lactic acid, the transcription level of *Hif-1α* was further elevated (Fig. 4J). By utilizing H4K12La antibodies to pull down protein-DNA complexes and performing de-crosslinking experiments, we discovered that the *Hif-1α* DNA content in the PA-treated group was significantly higher than that in the control group. The lactic acid and PA combination group had even higher *Hif-1α* DNA content. Notably, there was no significant difference in *iNOS* DNA content between the three groups (Fig. 4K). These findings suggest that lactic acid facilitates the transcription of *Hif-1α* by augmenting H4K12La levels, ultimately potentiating the inflammatory response induced by PA in macrophages.

### VB124 improves cardiac injury and reduces inflammatory macrophage infiltration in type 2 diabetic mice

**Fig. 5:**
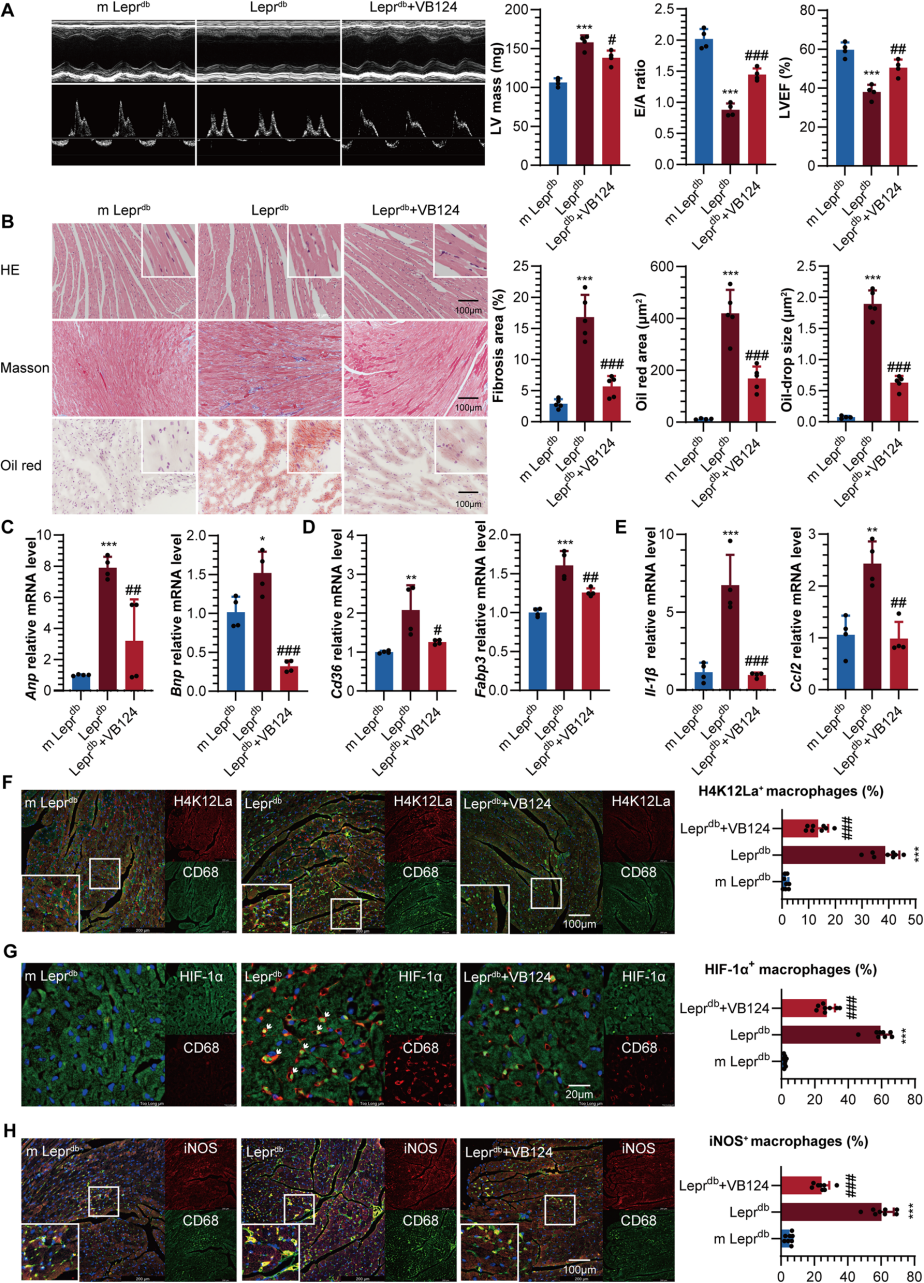
*Lepr^db^ compared to m Lepr^db^, ^#^Lepr^db^ + VB124 compared to Lepr^db^. **A** Pulsed‐wave Doppler showing: LV mass, E/A ratio and LVEF%. **B** Representative diagram and quantification of cardiac hypertrophy, fibrosis and oil-drop deposition in mice. **C** mRNA expression of cardiac *Anp* and *Bnp* in mice. **D** mRNA expression of cardiac *Cd36* and *Fabp3* in mice. **E** mRNA expression of cardiac *Il-1β* and *Ccl2* in mice. **F** Representative diagram and quantification of heart H4K12La positive macrophage (%) in mice. **G** Representative diagram and quantification of heart HIF-1α positive macrophage (%) in mice. **H** Representative diagram and quantification of heart iNOS positive macrophage (%) in mice

After a 4-week treatment with VB124, administered intraperitoneally at a dosage of 10 mg/kg/daily, a notable reduction in the left ventricular mass (LV mass) was observed in Lepr^db^ mice, accompanied by an increase in both the E/A ratio and left ventricular ejection fraction (LVEF) as illustrated in Fig. 5A. These data unequivocally establish that VB124 can significantly benefit cardiac hypertrophy and diastolic and systolic functions in type 2 diabetic mice. Pathological staining further revealed that VB124 treatment substantially reduced myocardial hypertrophy, interstitial fibrosis, and lipid droplet deposition in the Lepr^db^ mice (Fig. 5B, Additional file 1: Fig. S5A). This observation was further corroborated by RT-qPCR analysis, which demonstrated a significant downregulation of the transcription levels of *Anp* and *Bnp*, markers of myocardial injury in Lepr^db^ mice, following VB124 treatment (Fig. 5C). Moreover, VB124 was also found to significantly decrease the transcription levels of *Cd36* and fatty acid-binding protein 3 (*Fabp3*), key players in fatty acid transport (Fig. 5D), further highlighting the critical role of VB124 in ameliorating cardiac lipid droplet deposition.

Importantly, our study also revealed that VB124 treatment led to a marked reduction in ROS levels in the hearts of Lepr^db^ mice, concomitant with an increase in double-stranded DNA (dsDNA) content within mitochondrial compartments (Additional file 1: Fig. S5B). This provides direct evidence of improved mitochondrial function in the heart. Additionally, cytokine profiling revealed a significant downregulation of inflammatory mediators *Il-1β* and *Ccl2* transcription levels in Lepr^db^ mice receiving VB124 treatment (Fig. 5E), further corroborating the anti-inflammatory properties of VB124. Finally, through macrophage analysis, we observed a notable decrease in the infiltration of H4K12La, HIF-1α, iNOS, and IL-1β-positive macrophages in the hearts of Lepr^db^ mice treated with VB124 (Fig. 5F–H, Additional file 1: Fig. S5D). This finding not only reinforces the anti-inflammatory effects of VB124 but also sheds light on a potential mechanism underlying its cardioprotective effects through modulation of macrophage activity.

### Correlation between blood lactate concentration and cardiac injury in T2DM and its application in a predictive model for diastolic dysfunction

We gathered comprehensive information, medical histories, and medication usage from patients with clearly diagnosed T2DM. These patients were then divided into two groups based on their peripheral blood lactate concentrations: the normal lactate group (Lac < 2.2 mmol/L) and the elevated lactate group (Lac ≥ 2.2 mmol/L). A comparative analysis of the baseline data between these two groups and logistic regression analysis revealed gender to be a significant independent factor influencing hyperlactatemia in T2DM patients. Specifically, women were found to have a significantly lower risk of developing hyperlactatemia compared to men (Additional file 2: Tables S1, S2). Furthermore, an examination of laboratory test indicators and linear regression analysis identified blood glucose and triglycerides as independent factors influencing peripheral blood lactate concentrations, with a notably positive correlation observed (Additional file 2: Tables S3, S4). A more detailed comparison of cardiac observation indicators between the two groups, along with correlation analysis, highlighted a significant negative correlation between CK-MB, hs-TNT, NT-proBNP, MYO, LVDd, and the E/A ratio with peripheral blood lactate concentrations (Additional file 2: Table S6). These negative correlations remained consistent even after adjusting for confounding variables such as gender and diabetes duration (Additional file 2: Table S7).

**Fig. 6:**
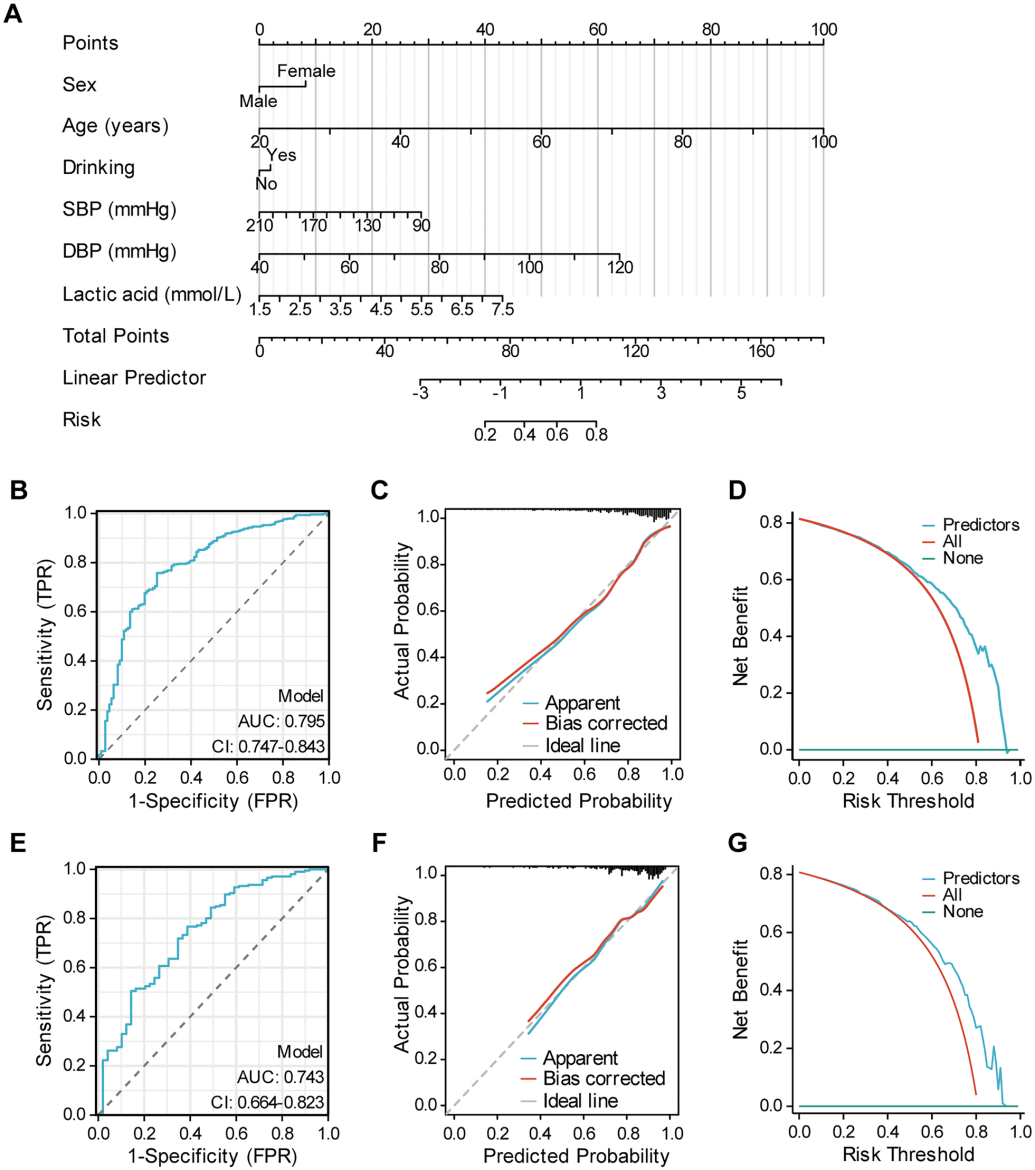
**A** Nomogram for the prediction of diastolic function in patients with T2DM. **B** ROC curve of training set, *ROC* receiver operating characteristic, *AUC* area under the ROC curve. **C** Calibration curve for predicting probability of diastolic function in patients with T2DM in training set. **D** Decision curve analysis in prediction of diastolic function in patients with T2DM in training set. **E** ROC curve of validation set. **F** Calibration curve for predicting probability of diastolic function in patients with T2DM in validation set. **G** Decision curve analysis in prediction of diastolic function in patients with T2DM in the validation set

Utilizing the aforementioned clinical dataset, we developed a clinical prediction nomogram specifically tailored for T2DM patients with cardiac diastolic dysfunction. This nomogram was constructed based on a training dataset comprising 599 T2DM patients and was externally validated using an independent dataset of 299 patients. A statistical analysis of the two datasets revealed no significant differences in most variable factors, except the proportion of sulfonylurea and insulin usage (Additional file 2: Table S8). A comprehensive statistical and logistic regression analysis of the general characteristic variables within the training dataset identified gender, age, alcohol consumption, systolic blood pressure, diastolic blood pressure, and lactate as independent predictors of T2DM with cardiac diastolic dysfunction (Additional file 2: Table S9). Leveraging these predictive indicators, we constructed a predictive nomogram for T2DM with cardiac diastolic dysfunction (Fig. 6A). This model demonstrated impressive discriminatory power, with an AUC (area under the ROC curve) of 0.7795 (95% CI 0.747–0.843), a sensitivity of 0.758, and a specificity of 0.748 (Fig. 6B). Additionally, our model exhibited strong goodness-of-fit, as evidenced by the close alignment of the nomogram’s calibration curve with the ideal value (Fig. 6C), and the decision curve analysis further highlighted its significant net benefit (Fig. 6D). Finally, we substantiated the reliability of our predictive model through rigorous external validation (Fig. 6E–G). These findings underscore the profound relationship between lactate and cardiac injury in T2DM and establish lactate as a robust independent predictor for assessing cardiac diastolic dysfunction. From a different perspective, this result reinforces the crucial role of regulating lactate transport in safeguarding the hearts of patients with T2DM from injury.

## Discussion and conclusion

**Fig. 7:**
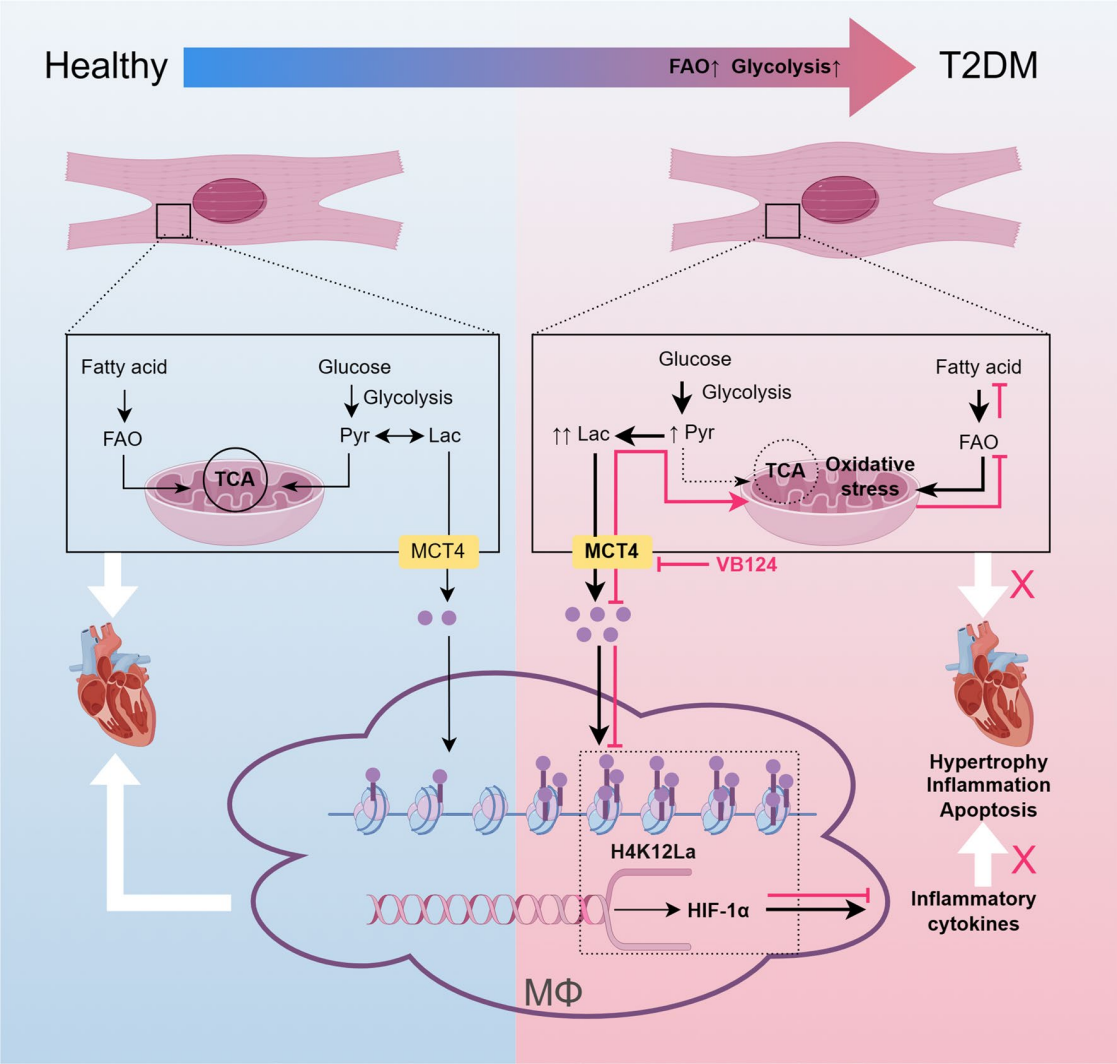
Mechanism of MCT4-dependent lactate transport-mediated diabetic cardiomyopathy

Although early studies have established a positive correlation between lactate and metabolic disorders in T2DM [18, 19], the role of lactate in human cardiac energy supply has only recently gained attention [20]. Nonetheless, the precise effects of elevated lactate on the heart in T2DM remain largely unexplored. In this study, we successfully developed a mouse model of type 2 diabetic cardiomyopathy that recapitulates metabolic hallmarks such as obesity, hyperglycemia, hyperlipidemia, and hyperlactatemia, along with pathological features including diastolic dysfunction and myocardial fibrosis. This model provides a robust tool for further investigations. Within this model, we uncovered the pivotal role of the gene *Slc16a3* and its encoded protein MCT4. Our findings elucidate the mechanism underlying fatty acid overload and lipotoxicity-induced T2DM myocardial injury, focusing on the involvement of the lactate-pyruvate axis and MCT4’s mediation of this process. Moreover, we demonstrated that inhibiting or knocking down MCT4 can reverse cardiac hypertrophy and apoptosis, attenuate the transcription of inflammatory cytokines, and potentially alleviate myocardial lipotoxicity. Additionally, we investigated the impact of upregulated MCT4 in cardiomyocytes on macrophages in a high-free fatty acid environment and how lactate influences macrophage pro-inflammatory polarization. Furthermore, our study confirms the protective effects of VB124 treatment on cardiac function in type 2 diabetic mice, potentially exerting its anti-inflammatory effects through macrophage modulation (Fig. 7). Finally, we established a strong correlation between peripheral blood lactate levels and cardiac diastolic dysfunction in T2DM patients, suggesting the potential of lactate as an independent predictor for cardiac diastolic dysfunction. These findings offer crucial insights into the pathogenesis of DCM and pave the way for developing innovative therapeutic strategies.

The alteration in myocardial cell energy metabolism is intimately associated with myocardial pathological injury [21–23]. Mature myocardial cells preferentially utilize fatty acids as an energy source due to the greater energy efficiency of fatty acid oxidation. Nevertheless, myocardial cells augment glycolysis activity under hypoxic or ischemic conditions to generate additional ATP and sustain normal cardiac function. This metabolic adaptation caters to the heart’s exorbitant energy demands and mitigates oxidative stress by reducing free radicals produced during fatty acid metabolism. However, in our observations of Lepr^db^ mice fed a high-fat diet, we observed concurrent upregulation of cardiac fatty acid oxidation and glycolysis pathways, inevitably culminating in cellular oxidative stress damage and lactate accumulation. We substantiated this inference through experiments on myocardial cells. Nonetheless, the precise implications of lactate accumulation on myocardial metabolic injury warrant further elucidation.

Recent studies have suggested that glucose fuels the TCA cycle with energy derived from circulating lactate, highlighting the significance of lactate as a crucial energy source for the heart [24]. The conversion of lactate into pyruvate, catalyzed by lactate dehydrogenase (LDH), precedes its transport into the mitochondrial matrix via the mitochondrial pyruvate carrier (MPC). Subsequently, pyruvate enters the TCA cycle, leading to ATP production. Furthermore, malonyl-CoA, a byproduct of lactate metabolism, can attenuate mitochondrial free fatty acid derivative uptake by inhibiting CPT1, thus mitigating excessive fatty acid oxidation [25]. Accumulation of lactate and pyruvate may also contribute to reduced intracellular reactive oxygen species levels, possibly due to their capacity to scavenge free radicals [26, 27]. Therefore, lactate can function as a direct energy source and a potential protective agent in myocardial cells. Consequently, the augmented glycolysis and lactate accumulation observed in Lepr^db^ mice fed a high-fat diet may represent a compensatory response to excessive fatty acid oxidation, aiding in mitigating lipid overload and oxidative stress-induced damage in myocardial cells.

The monocarboxylate transporter MCT4 is pivotal in transporting lactate from cells to the extracellular space in a high-capacity, low-affinity manner [28]. As such, its significance in lactate metabolism and regulation cannot be disregarded. Typically, MCT4 expression remains low in most cell types, including myocardial cells. However, its expression is upregulated in response to myocardial damage or perfusion with lactate [29, 30]. It has been reported that knocking out CD147, a widely expressed plasma membrane glycoprotein essential for MCT4 function and localization, can alleviate myocardial injury resulting from pulmonary embolism and chronic pressure overload [31, 32]. Recently, a novel and effective MCT4 inhibitor has been introduced, which prevents and reverses cardiac hypertrophy in mice by regulating the lactate-pyruvate metabolic axis [16]. In our study utilizing a fatty acid-induced type 2 diabetic cardiomyopathy mouse model and myocardial cells, we observed upregulated MCT4 expression leading to increased lactate efflux from myocardial cells, disrupting the intracellular lactate-pyruvate axis and subsequently weakening the protective effects of intracellular lactate. Strategic knockdown or inhibition of MCT4 within the cardiomyocytes mitigates the efflux of lactic acid triggered by free fatty acids. It facilitates the conversion of lactic acid to pyruvic acid and its subsequent entry into the TCA cycle. This intervention holds promise in reversing mitochondrial oxidative stress injury, inflammation, and cell apoptosis, paving the way for potential therapeutic strategies to manage diabetic cardiomyopathy.

The role of lactic acid exhibits considerable variability across different cell types. For instance, tumor-derived lactic acid can suppress the immune response of tumor-associated macrophages and T cells against tumors, favoring the growth of cancer cells [33–35]. In contrast, during chronic or acute inflammation, the immune response to lactic acid differs markedly from that observed in tumors. Our findings align with Samuvel et al.’s findings, demonstrating that lactic acid can enhance Toll-like receptor 4 ligand-induced polarization of human macrophages towards the pro-inflammatory phenotype [36]. Furthermore, the accumulation of lactic acid in chronic inflammatory sites can promote rheumatoid arthritis by inducing metabolic reprogramming in CD4+ T cells [37]. Therefore, in healthy and type 2 diabetic conditions, where anti-inflammatory and pro-inflammatory signals dominate, lactic acid may exert distinct regulatory functions in the microenvironment. Macrophages, as inherent immune cells of the body, are particularly susceptible to epigenetic modifications influenced by the microenvironment. Previous studies have revealed a novel epigenetic modification induced by lactic acid-lactylation. This research indicates increased H3K18 lactylation in macrophages regulates gene expression, contributing to homeostatic processes like wound healing [11]. Similarly, studies have shown that H3K18 lactylation regulates the anti-inflammatory and pro-angiogenic activity of monocytes/macrophages by promoting the transcription of repair genes, leading to improved cardiac function after myocardial infarction [12]. However, other studies have demonstrated that H4K12 lactylation exacerbates microglial dysfunction in Alzheimer’s disease by activating a positive feedback loop involving glycolysis/H4K12La/ PKM2 through the transcription of genes such as *Ldha*, *Pkm2*, and *Hif-1α* [13]. These suggest that histone lactylation at different sites can lead to substantial variations in their regulatory functions. Our research indicates that lactic acid can simultaneously induce H3K18La and H4K12La in macrophages. However, in a high-free fatty acid environment, lactic acid primarily exacerbates H4K12La, mediating macrophage inflammation. In high-fat-induced type 2 diabetic mice, inhibiting MCT4 reverses cardiac macrophage H4K12La, alleviating cardiac inflammation. These findings highlight lactic acid’s complex and context-specific role in regulating immune responses and underscore the importance of considering these nuances in therapeutic interventions.

HIF-1α is a pivotal transcription factor activated in response to hypoxic conditions. It plays a crucial role in regulating the expression of a myriad of genes and actively participates in cellular stress responses [38]. In pathological states characterized by hypertension and obesity, the expression levels of HIF-1α in myocardial tissue macrophages are notably elevated. This upregulation mediates the release of pro-inflammatory factors, augments oxidative stress, and ultimately contributes to myocardial cell damage and the progression of myocardial pathology [9]. In our investigation, we have observed transcriptional activation of *Hif-1α* in macrophages induced by a high-fat environment, which is accompanied by upregulated expression of inflammatory cytokines such as* Il-1β*. Furthermore, our research has implicated H4K12La in the transcriptional activation of *Hif-1α*. Surprisingly, inhibiting MCT4 in high-fat-induced type 2 diabetic mice not only reduces the modification level of H4K12La in cardiac macrophages but also significantly decreases the infiltration of HIF-1α-positive and IL-1β-positive inflammatory macrophages in the heart. This suggests that the inhibition of MCT4 plays a significant role in alleviating cardiac inflammation in type 2 diabetic mice.

Previous studies have consistently reported elevated serum lactic acid levels in patients with obesity, hypertension, and insulin resistance, which are strongly correlated with fasting blood glucose, glycosylated hemoglobin, and the development of type 2 diabetes. A prospective study further substantiated that lactic acid is an independent risk factor for type 2 diabetes [18–20]. However, the precise relationship between circulating lactic acid levels and cardiac injury remains elusive. Therefore, we conducted a comprehensive clinical data analysis in patients with T2DM. Our observations revealed a notable association between serum lactic acid concentrations and markers of cardiac injury in type 2 diabetes, suggesting a potential predictive value for diastolic dysfunction. Based on these insights, we have formulated a clinical prediction model tailored for diastolic dysfunction in T2DM. Although our clinical study did not directly explore the role of cardiac lactic acid and its transport in DCM, it indirectly supports the plausibility of therapeutic strategies centered on lactic acid regulation for managing DCM.

Despite the notable findings of this study, some several limitations and shortcomings merit consideration. Firstly, in terms of animal experiment design, we primarily selected male mice as our research subjects. This choice was based on the assumption that male mice have a more stable physiological state than female mice, which helps to reduce experimental variables. However, this also means that our study did not adequately cover the female population, particularly in exploring the relationship between lactic acid and diabetic cardiomyopathy. Therefore, in future studies, we will emphasize the impact of gender factors on experimental results and strive to address this limitation. Secondly, although the study suggests a potential role of imbalanced lactic acid transport in DCM, techniques such as isotope labeling and metabolic flux detection were not employed to establish the directionality of lactic acid flow unequivocally. In addition, while we observed a direct correlation between lactic acid-induced H4K12 lactylation of macrophages and HIF-1α activation, accompanied by the detection of pertinent inflammatory markers, empirical evidence is currently lacking to elucidate the precise mechanism underlying HIF-1α’s regulation of macrophage inflammation. Finally, although this study demonstrated that VB124 exerted a protective effect on diabetic cardiomyopathy and observed a reduction in H4K12 activation of macrophages, further investigations are warranted to assess whether VB124 impacts other epigenetic modifications of macrophage histone. It is essential to acknowledge that the majority of this study was conducted using mouse and cell models, necessitating further validation in human subjects to confirm the relevance and applicability of the conclusions.

In summary, our comprehensive investigation has unveiled the pivotal role of MCT4-dependent lactic acid transport in mediating energy metabolism impairments and inflammatory responses in the hearts of type 2 diabetes, thereby confirming the protective effects of MCT4 inhibition in DCM. These groundbreaking findings offer profound insights into the underlying pathogenic mechanisms of DCM and establish a robust theoretical framework for advancing innovative therapeutic approaches.

### Supplementary Information

**Additional file 1: Figure S1.** A: GSEA enrichment analysis of DEGs in Lepr^db^ vs. m Lepr^db^ mice. B: CIBERSORT immune infiltration assessment of heart tissue in Lepr^db^ vs. m Lepr^db^ mice. C: PPI network and hub genes. D: Western blot analysis and quantification of MCT4 in the plasma membrane components of heart tissue in Lepr^db^ vs. m Lepr^db^ mice. **Figure S2.** A: Representative diagram of Λψm (mitochondrial membrane potential) in H9C2 cells treated with PA. B: Fold change of pyruvate in H9C2 whole cell lysates after time-dependent PA stimulation. C: Western blot analysis and quantification of MCT4 in the plasma membrane components of H9C2 cells after time-dependent PA stimulation. D: Fold change in whole-cell abundances of pyruvate in H9C2 treated with PA and/or VB124. E: Fold change in mitochondrial pool abundances of pyruvate in H9C2 treated with PA and/or VB124. F: Fold change in whole-cell abundances of lactic acid in H9C2 treated with PA and/or MCT4 knock-down (KD). G: Fold change in whole-cell abundances of pyruvate in H9C2 treated with PA and/or MCT4 KD. H: Fold change in mitochondrial pool abundances of pyruvate in H9C2 treated with PA and/or MCT4 KD. I: Fold change in cell supernatant abundances of lactic acid in H9C2 treated with PA and/or MCT4 KD. J: Representative diagram and quantification of ROS in H9C2 cells treated with PA and/or MCT4 KD. K: Representative diagram of MitoSOX in PMCM and H9C2 cells treated with PA and/or VB124. L: Representative diagram and quantification of Λψm in H9C2 cells treated with PA and/or MCT4 KD. M: Fold change of ATP content in H9C2 treated with PA and/or MCT4 KD. **Figure S3.** A: Representative diagram and quantification of BNP in H9C2 cells treated with PA and/or MCT4 KD. B: *Anp* and *Bnp* mRNA expression in H9C2 cells treated with PA and/or MCT4 KD. C: Representative diagram and quantification of length–width ratio in H9C2 cells treated with PA and/or VB124. D: Representative diagram and quantification of Annexin-V positive apoptosis cells in H9C2 treated with PA and/or VB124. E: mRNA expression of *Il-1β, Il-6, Il-18, Ccl2* and *Tnf-α* in H9C2 cells treated with PA and/or MCT4 KD. **Figure S4.** RAW 264.7 and H9C2 (pre-treated with PA and/or MCT4 KD) were cocultured for 24 h for A–C. A: Representative diagram and quantification of migrated RAW 264.7 cells stained with crystal violet. B: mRNA expression of *Il-1β* and *Tnf-α* in coculture RAW 264.7. C: mRNA expression of *iNOS, Hif-1α*, *Il-1β*, and *Tnf-α* in coculture RAW 264.7. D: Representative diagram and quantification of ARG-1 and iNOS in RAW 264.7 treated with PA and/or LAC. E: Fold change in whole-cell abundances of lactic acid in RAW 264.7 treated with PA and/or LAC. F: mRNA expression of *Il-6* and *Il-18* in RAW 264.7 treated with PA and/or LAC. **Figure S5.** A: Representative diagram of heart oil-drop deposition in mice. B: Representative diagram and quantification of heart ROS and mitochondria dsDNA/total cytoplasmic dsDNA in mice. C: mRNA expression of cardiac *Il-6* and *Il-18* in mice. D: Representative diagram and quantification of heart IL-1β positive macrophage (%) in mice. *Lepr^db^ compared to m Lepr^db^, ^#^Lepr^db^ + VB124 compared to Lepr^db^.

**Additional file 2: Table S1.** Comparison of baseline between two groups. **Table S2.** Single and multiple logistic regression analysis. **Table S3.** Comparison of general indexes between two groups. **Table S4.** Linear regression analysis of metabolic factors affecting lactic acid. **Table S5.** Comparison of the results of cardiac indicators between two groups. **Table S6.** Correlation analysis between blood lactic acid level and cardiac indicators. **Table S7.** Baseline characteristics of all patients in the training set and validation set. **Table S8.** General characteristics of the patients and logistic regression analyses for screening predictors.

## Data Availability

The datasets generated and/or analyzed in the current study are available from the corresponding author upon reasonable request.

## References

[1] Gulsin GS, Athithan L, McCann GP (2019). Diabetic cardiomyopathy: prevalence, determinants and potential treatments. Ther Adv Endocrinol Metab.

[2] Peterson LR, Gropler RJ (2020). Metabolic and molecular imaging of the diabetic cardiomyopathy. Circ Res.

[3] Tan Y, Zhang Z, Zheng C, Wintergerst KA, Keller BB, Cai L (2020). Mechanisms of diabetic cardiomyopathy and potential therapeutic strategies: preclinical and clinical evidence. Nat Rev Cardiol.

[4] Brooks GA, Arevalo JA, Osmond AD, Leija RG, Curl CC, Tovar AP (2022). Lactate in contemporary biology: a phoenix risen. J Physiol.

[5] Li X, Yang Y, Zhang B (2022). Lactate metabolism in human health and disease. Signal Transduct Target Ther.

[6] Dong S, Qian L, Cheng Z (2021). Lactate and myocardiac energy metabolism. Front Physiol.

[7] Brooks GA (2018). The science and translation of lactate shuttle theory. Cell Metab.

[8] Swirski FK, Nahrendorf M (2018). Cardioimmunology: the immune system in cardiac homeostasis and disease. Nat Rev Immunol.

[9] Mouton AJ, Li X, Hall ME, Hall JE (2020). Obesity, hypertension, and cardiac dysfunction: novel roles of immunometabolism in macrophage activation and inflammation. Circ Res.

[10] Frantz S, Falcao-Pires I, Balligand JL (2018). The innate immune system in chronic cardiomyopathy: a European Society of Cardiology (ESC) scientific statement from the Working Group on Myocardial Function of the ESC. Eur J Heart Fail.

[11] Zhang D, Tang Z, Huang H (2019). Metabolic regulation of gene expression by histone lactylation. Nature.

[12] Wang N, Wang W, Wang X (2022). Histone lactylation boosts reparative gene activation post-myocardial infarction. Circ Res.

[13] Pan RY, He L, Zhang J (2022). Positive feedback regulation of microglial glucose metabolism by histone H4 lysine 12 lactylation in Alzheimer’s disease. Cell Metab.

[14] Ma XM, Geng K, Law BY (2023). Lipotoxicity-induced mtDNA release promotes diabetic cardiomyopathy by activating the cGAS-STING pathway in obesity-related diabetes. Cell Biol Toxicol.

[15] Ussher JR (2014). The role of cardiac lipotoxicity in the pathogenesis of diabetic cardiomyopathy. Expert Rev Cardiovasc Ther.

[16] Cluntun AA, Badolia R, Lettlova S (2021). The pyruvate-lactate axis modulates cardiac hypertrophy and heart failure. Cell Metab.

[17] Bisetto S, Wright MC, Nowak RA (2019). New insights into the lactate shuttle: role of MCT4 in the modulation of the exercise capacity. iScience.

[18] Chen YD, Varasteh BB, Reaven GM (1993). Plasma lactate concentration in obesity and type 2 diabetes. Diabete Metab.

[19] Ohlson LO, Larsson B, Björntorp P (1988). Risk factors for type 2 (non-insulin-dependent) diabetes mellitus. Thirteen and one-half years of follow-up of the participants in a study of Swedish men born in 1913. Diabetologia.

[20] Murashige D, Jang C, Neinast M (2020). Comprehensive quantification of fuel use by the failing and nonfailing human heart. Science.

[21] Tang X (2023). Regenerating the heart by metabolically reprogramming the cardiomyocyte epigenome. Cell Metab.

[22] Mohamed TMA, Abouleisa R, Hill BG (2022). Metabolic determinants of cardiomyocyte proliferation. Stem Cells.

[23] Serio S, Pagiatakis C, Musolino E (2023). Cardiac aging is promoted by pseudohypoxia increasing p300-induced glycolysis. Circ Res.

[24] Hui S, Ghergurovich JM, Morscher RJ (2017). Glucose feeds the TCA cycle via circulating lactate. Nature.

[25] Ngo J, Choi DW, Stanley IA (2023). Mitochondrial morphology controls fatty acid utilization by changing CPT1 sensitivity to malonyl-CoA. EMBO J.

[26] Liu X, Cooper DE, Cluntun AA (2018). Acetate production from glucose and coupling to mitochondrial metabolism in mammals. Cell.

[27] Groussard C, Morel I, Chevanne M, Monnier M, Cillard J, Delamarche A (2000). Free radical scavenging and antioxidant effects of lactate ion: an in vitro study. J Appl Physiol.

[28] Felmlee MA, Jones RS, Rodriguez-Cruz V, Follman KE, Morris ME (2020). Monocarboxylate transporters (SLC16): function, regulation, and role in health and disease. Pharmacol Rev.

[29] Zhu Y, Wu J, Yuan SY (2013). MCT1 and MCT4 expression during myocardial ischemic-reperfusion injury in the isolated rat heart. Cell Physiol Biochem.

[30] Gabriel-Costa D, Cunha TF, Paixão NA (2018). Lactate-upregulation of lactate oxidation complex-related genes is blunted in left ventricle of myocardial infarcted rats. Braz J Med Biol Res.

[31] Lu G, Jia Z, Zu Q, Zhang J, Zhao L, Shi H (2018). Inhibition of the cyclophilin A-CD147 interaction attenuates right ventricular injury and dysfunction after acute pulmonary embolism in rats. J Biol Chem.

[32] Suzuki K, Satoh K, Ikeda S (2016). Basigin promotes cardiac fibrosis and failure in response to chronic pressure overload in mice. Arterioscler Thromb Vasc Biol.

[33] Bohn T, Rapp S, Luther N (2018). Tumor immunoevasion via acidosis-dependent induction of regulatory tumor-associated macrophages. Nat Immunol.

[34] Colegio OR, Chu NQ, Szabo AL (2014). Functional polarization of tumour-associated macrophages by tumour-derived lactic acid. Nature.

[35] Reina-Campos M, Moscat J, Diaz-Meco M (2017). Metabolism shapes the tumor microenvironment. Curr Opin Cell Biol.

[36] Samuvel DJ, Sundararaj KP, Nareika A, Lopes-Virella MF, Huang Y (2009). Lactate boosts TLR4 signaling and NF-kappaB pathway-mediated gene transcription in macrophages via monocarboxylate transporters and MD-2 up-regulation. J Immunol.

[37] Pucino V, Certo M, Bulusu V (2019). Lactate buildup at the site of chronic inflammation promotes disease by inducing CD4+ T cell metabolic rewiring. Cell Metab.

[38] Sant'Ana PG, Tomasi LC, Murata GM (2023). Hypoxia-inducible factor 1-alpha and glucose metabolism during cardiac remodeling progression from hypertrophy to heart failure. Int J Mol Sci.

