# Supplementary material for: MCT4-dependent lactate transport: a novel mechanism for cardiac energy metabolism injury and inflammation in type 2 diabetes mellitus
Source: Cardiovasc Diabetol. 2024 Mar 14;23:96. doi: 10.1186/s12933-024-02178-2 (PMC10941417; doi:10.1186/s12933-024-02178-2)
Supplement: Supplementary file 1 — Additional file 1: Figure S1. A: GSEA enrichment analysis of DEGs in Leprdb vs. m Leprdb mice. B: CIBERSORT immune infiltration assessment of heart tissue in Leprdb vs. m Leprdb mice. C: PPI network and hub genes. D: Western blot analysis and quantification of MCT4 in the plasma membrane components of heart tissue in Leprdb vs. m Leprdb mice. Figure S2. A: Representative diagram of Λψm (mitochondrial membrane potential) in H9C2 cells treated with PA. B: Fold change of pyruvate in H9C2 whole cell lysates after time-dependent PA stimulation. C: Western blot analysis and quantification of MCT4 in the plasma membrane components of H9C2 cells after time-dependent PA stimulation. D: Fold change in whole-cell abundances of pyruvate in H9C2 treated with PA and/or VB124. E: Fold change in mitochondrial pool abundances of pyruvate in H9C2 treated with PA and/or VB124. F: Fold change in whole-cell abundances of lactic acid in H9C2 treated with PA and/or MCT4 knock-down (KD). G: Fold change in whole-cell abundances of pyruvate in H9C2 treated with PA and/or MCT4 KD. H: Fold change in mitochondrial pool abundances of pyruvate in H9C2 treated with PA and/or MCT4 KD. I: Fold change in cell supernatant abundances of lactic acid in H9C2 treated with PA and/or MCT4 KD. J: Representative diagram and quantification of ROS in H9C2 cells treated with PA and/or MCT4 KD. K: Representative diagram of MitoSOX in PMCM and H9C2 cells treated with PA and/or VB124. L: Representative diagram and quantification of Λψm in H9C2 cells treated with PA and/or MCT4 KD. M: Fold change of ATP content in H9C2 treated with PA and/or MCT4 KD. Figure S3. A: Representative diagram and quantification of BNP in H9C2 cells treated with PA and/or MCT4 KD. B: Anp and Bnp mRNA expression in H9C2 cells treated with PA and/or MCT4 KD. C: Representative diagram and quantification of length–width ratio in H9C2 cells treated with PA and/or VB124. D: Representative diagram and quantification of Annexin-V positive apoptosi [file 12933_2024_2178_MOESM1_ESM.docx]

**Additional Figures**


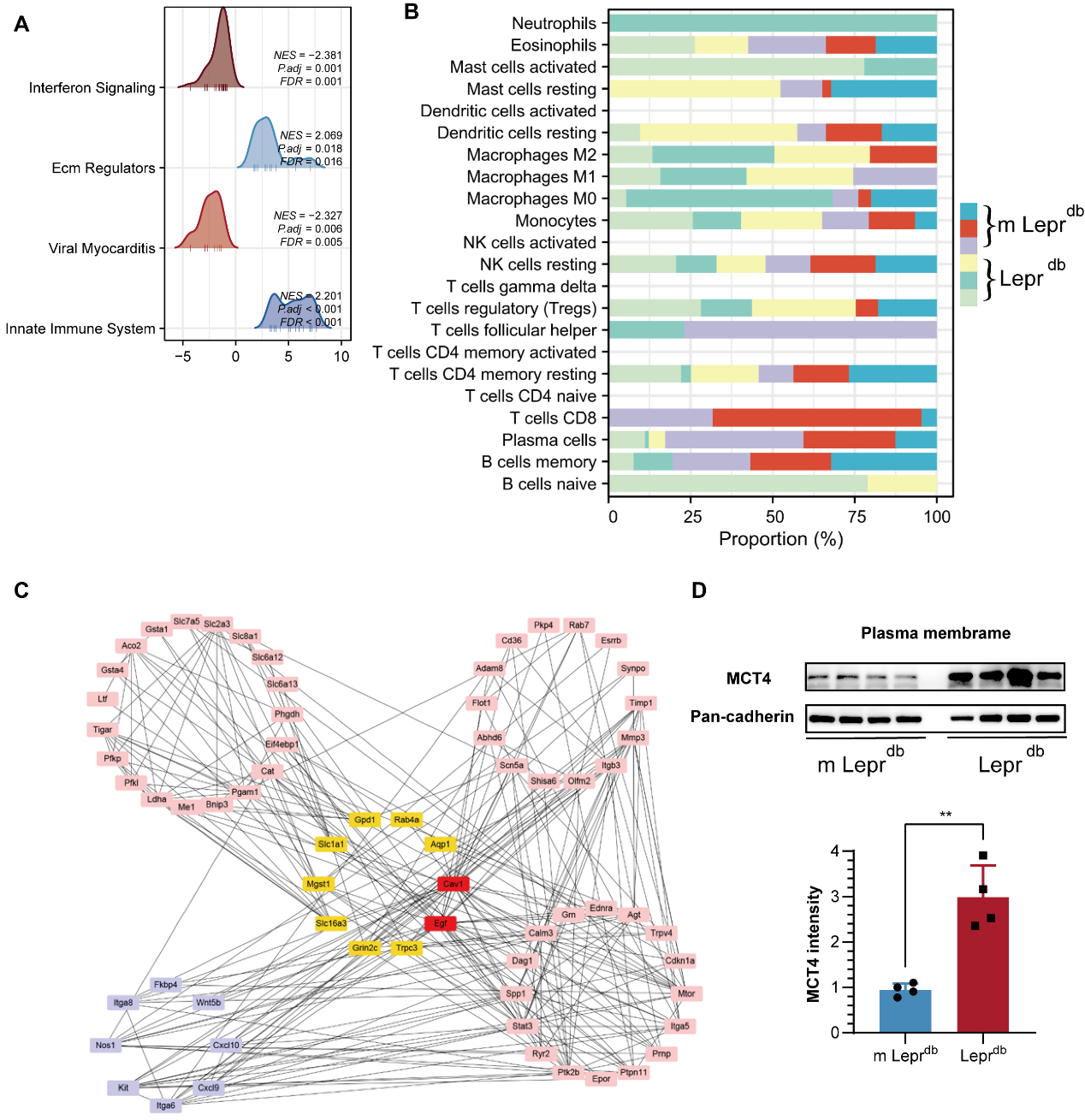


**Figure S1** A: GSEA enrichment analysis of DEGs in Lepr^db^ vs. m Lepr^db^ mice. B: CIBERSORT immune infiltration assessment of heart tissue in Lepr^db^ vs. m Lepr^db^ mice. C: PPI network and hub genes. D: Western blot analysis and quantification of MCT4 in the plasma membrane components of heart tissue in Lepr^db^ vs. m Lepr^db^ mice.


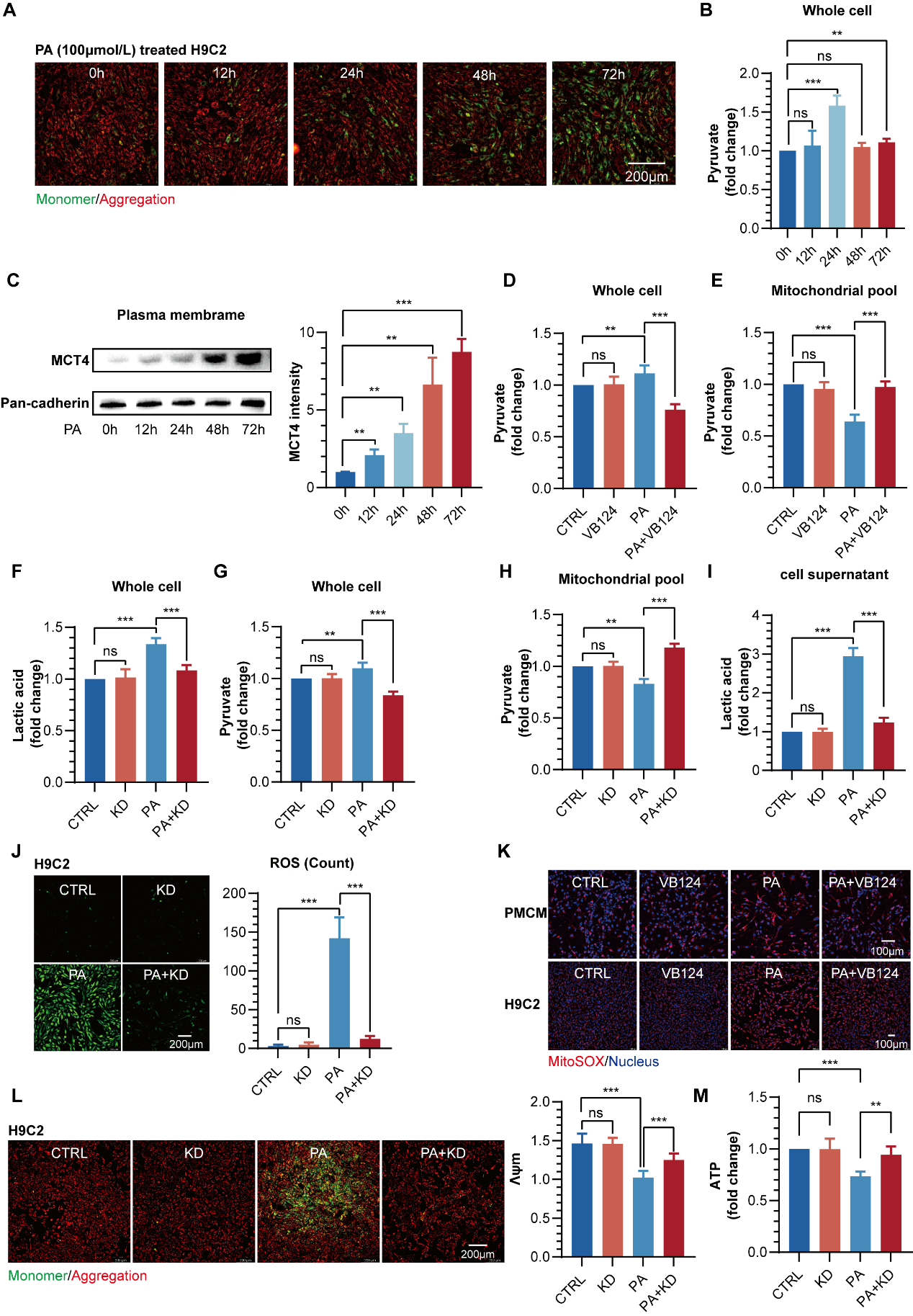


**Figure S2** A: Representative diagram of Λψm (mitochondrial membrane potential) in H9C2 cells treated with PA. B: Fold change of pyruvate in H9C2 whole cell lysates after time-dependent PA stimulation. C: Western blot analysis and quantification of MCT4 in the plasma membrane components of H9C2 cells after time-dependent PA stimulation. D: Fold change in whole-cell abundances of pyruvate in H9C2 treated with PA and/or VB124. E: Fold change in mitochondrial pool abundances of pyruvate in H9C2 treated with PA and/or VB124. F: Fold change in whole-cell abundances of lactic acid in H9C2 treated with PA and/or MCT4 knock-down (KD). G: Fold change in whole-cell abundances of pyruvate in H9C2 treated with PA and/or MCT4 KD. H: Fold change in mitochondrial pool abundances of pyruvate in H9C2 treated with PA and/or MCT4 KD. I: Fold change in cell supernatant abundances of lactic acid in H9C2 treated with PA and/or MCT4 KD. J: Representative diagram and quantification of ROS in H9C2 cells treated with PA and/or MCT4 KD. K: Representative diagram of MitoSOX in PMCM and H9C2 cells treated with PA and/or VB124. L: Representative diagram and quantification of Λψm in H9C2 cells treated with PA and/or MCT4 KD. M: Fold change of ATP content in H9C2 treated with PA and/or MCT4 KD.


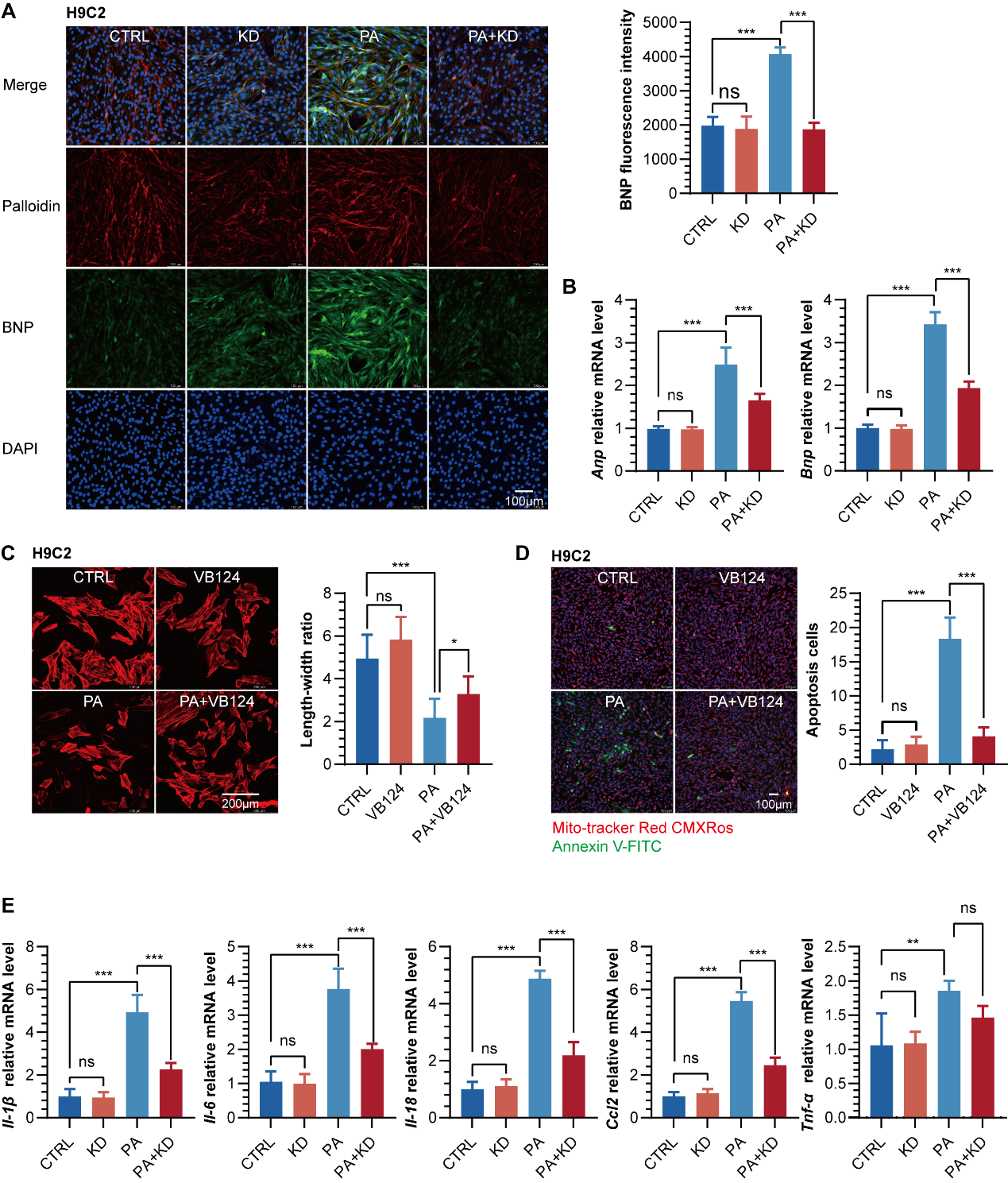


**Figure S3** A: Representative diagram and quantification of BNP in H9C2 cells treated with PA and/or MCT4 KD. B: *Anp* and *Bnp* mRNA expression in H9C2 cells treated with PA and/or MCT4 KD. C: Representative diagram and quantification of length-width ratio in H9C2 cells treated with PA and/or VB124. D: Representative diagram and quantification of Annexin-V positive apoptosis cells in H9C2 treated with PA and/or VB124. E: mRNA expression of *Il-1β, Il-6, Il-18, Ccl2* and *Tnf-α* in H9C2 cells treated with PA and/or MCT4 KD.


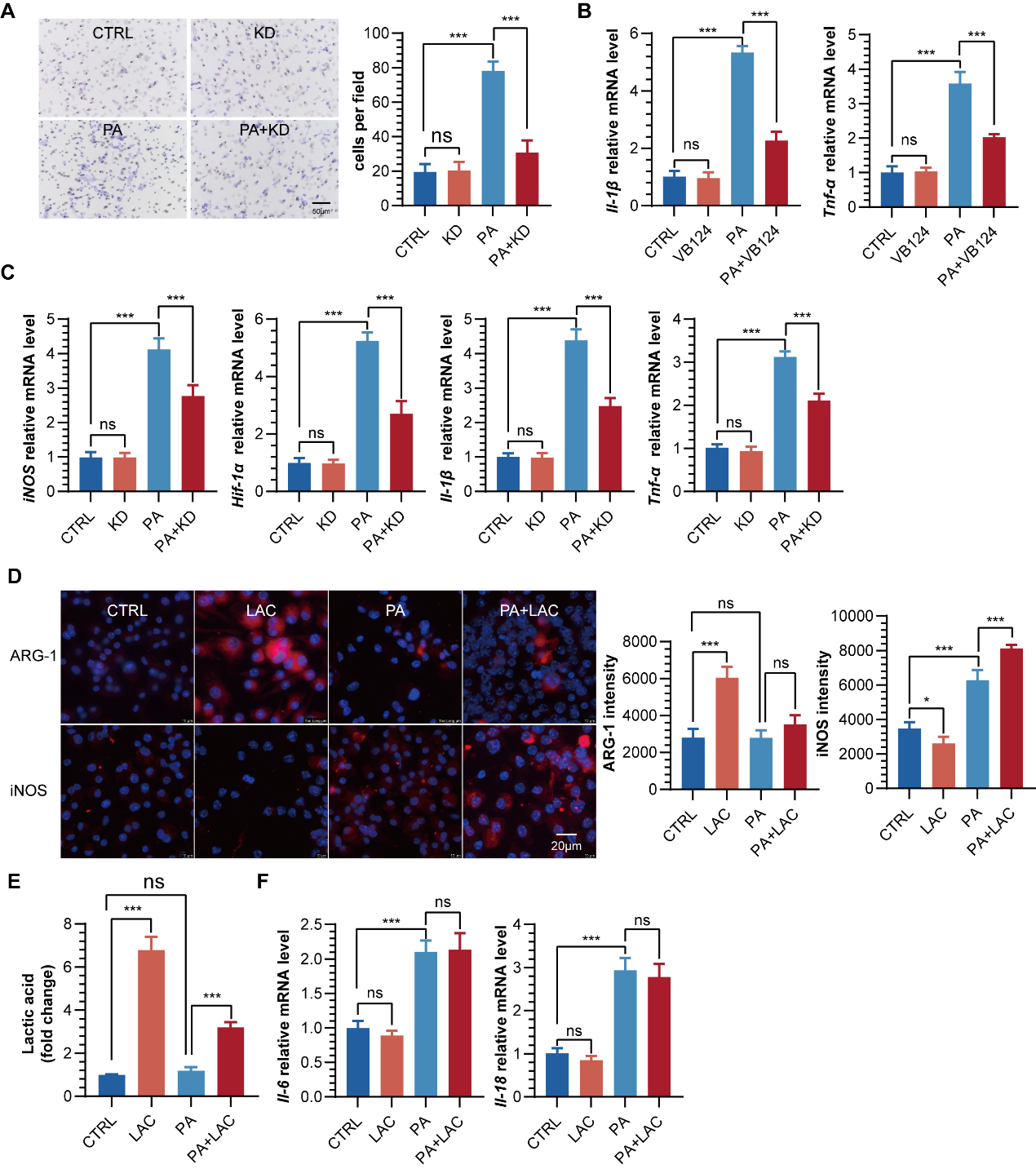


**Figure S4** RAW 264.7 and H9C2 (pre-treated with PA and/or MCT4 KD) were cocultured for 24h for A-C. A: Representative diagram and quantification of migrated RAW 264.7 cells stained with crystal violet. B: mRNA expression of *Il-1β* and *Tnf-α* in coculture RAW 264.7. C: mRNA expression of *iNOS, Hif-1α, Il-1β,* and *Tnf-α* in coculture RAW 264.7. D: Representative diagram and quantification of ARG-1 and iNOS in RAW 264.7 treated with PA and/or LAC. E: Fold change in whole-cell abundances of lactic acid in RAW 264.7 treated with PA and/or LAC. F: mRNA expression of *Il-6* and *Il-18* in RAW 264.7 treated with PA and/or LAC.


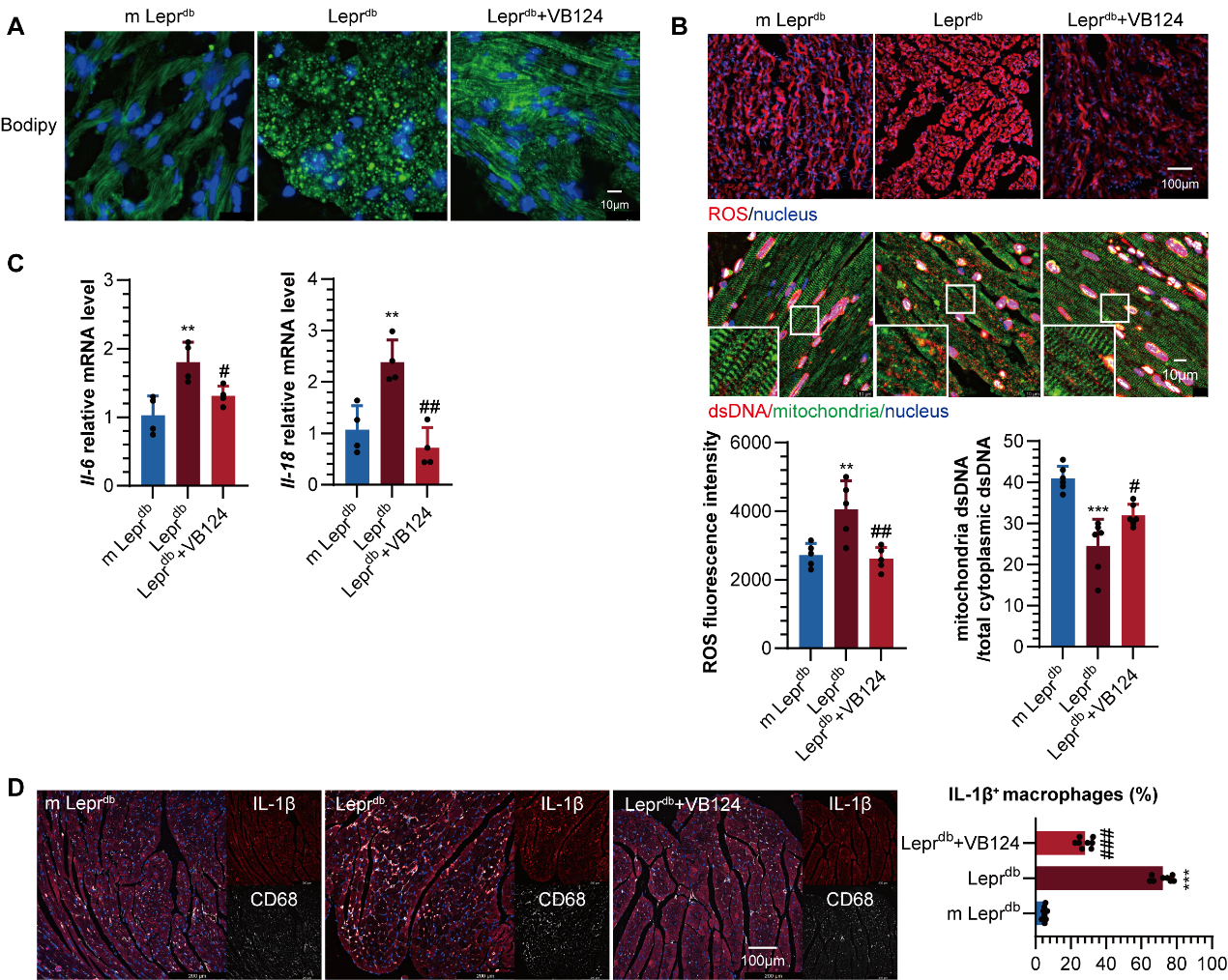


**Figure S5** A: Representative diagram of heart oil-drop deposition in mice. B: Representative diagram and quantification of heart ROS and mitochondria dsDNA/ total cytoplasmic dsDNA in mice. C: mRNA expression of cardiac *Il-6* and *Il-18* in mice. D: Representative diagram and quantification of heart IL-1β positive macrophage (%) in mice. * Lepr^db^ compared to m Lepr^db^, # Lepr^db^+VB124 compared to Lepr^db^.
