# Supplementary material for: MCT4-dependent lactate transport: a novel mechanism for cardiac energy metabolism injury and inflammation in type 2 diabetes mellitus
Source: Cardiovasc Diabetol. 2024 Mar 14;23:96. doi: 10.1186/s12933-024-02178-2 (PMC10941417; doi:10.1186/s12933-024-02178-2)
Supplement: Supplementary file 2 — Additional file 2: Table S1. Comparison of baseline between two groups. Table S2. Single and multiple logistic regression analysis. Table S3. Comparison of general indexes between two groups. Table S4. Linear regression analysis of metabolic factors affecting lactic acid. Table S5. Comparison of the results of cardiac indicators between two groups. Table S6. Correlation analysis between blood lactic acid level and cardiac indicators. Table S7. Baseline characteristics of all patients in the training set and validation set. Table S8. General characteristics of the patients and logistic regression analyses for screening predictors. [file 12933_2024_2178_MOESM2_ESM.docx]

**Table S1 Comparison of baseline between two groups**

|  | T2DM with  Lac＜2.2mmol/L  n=797 | T2DM with  Lac＞=2.2mmol/L  n=797 | *P*value |
| --- | --- | --- | --- |
| Sex |  |  |  |
| Male（%） | 142（47.3%） | 457（57.3%） | **0.003^*^** |
| Female（%） | 158（52.7%） | 340（42.7%） |  |
| Age（year） | 63（54-70） | 61（53-69） | 0.262 |
| SBP（mm/Hg） | 138（125-154.5） | 136.5（123-150.5） | 0.065 |
| DBP（mm/Hg） | 78（71-86.5） | 78（71-86） | 0.709 |
| T2DM duration |  |  | 0.003* |
| Newly新 | 13（4.3%） | 62（7.8%） |  |
| ＜1 year | 7（2.3%） | 56（7.0%） |  |
| 1~3 year | 28（9.3%） | 51（6.4%） |  |
| 3~5 year | 25（8.3%） | 77（9.7%） |  |
| 5~10 years | 53（17.7%） | 150（18.8%） |  |
| ＞10 years | 174（58.0%） | 401（50.3%） |  |
| Smoking（%） | 67（22.3%） | 220（27.6%） | 0.077 |
| Drinking（%） | 81（27.0%） | 260（32.6%） | 0.073 |
| Anti-diabetes drugs use |  |  |  |
| Metformin二 | 207 (69%) | 563 (70.5%) | 0.637 |
| Sulfonylurea磺 | 113（38.4%） | 299（40.7%） | 0.507 |
| Glinides格 | 9（3.1%） | 21（2.9%） | 0.860 |
| Thiazolidinediones噻 | 19（6.5%） | 55（7.5%） | 0.567 |
| α-glucosidase inhibitor | 53（18.0%） | 172（23.4%） | 0.060 |
| SGLT2 inhibitor抑 | 29（9.9%） | 85（11.6%） | 0.432 |
| DPP-4 inhibitor抑 | 24（8.2%） | 70（9.5%） | 0.494 |
| GLP-1 agonist激 | 3（1.0%） | 20（2.7%） | 0.095 |
| Insulin胰 | 154（52.4%） | 331（45.0%） | 0.033^*^ |

SBP: Systolic blood pressure; DBP: Diastolic blood pressure; SGLT2 inhibitor: Sodium-glucose co-transporter 2 inhibitor; DPP4 inhibitor: Dipeptidyl peptidase-4 inhibitor; GLP-1 agonist: Glucagon-like peptide-1 agonist.

**Table S2 Single and multiple logistic regression analysis**

| **Varies** | **Univariate analysis** | |  | **Multivariate analysis** | |
| --- | --- | --- | --- | --- | --- |
|  | Odds Ratio (95% CI) | P value |  | Odds Ratio (95% CI) | P value |
| Sex |  |  |  |  |  |
| Male | Reference |  |  | Reference |  |
| Female | 0.669 (0.513 — 0.873) | **0.003** |  | 0.670 (0.464 — 0.967) | **0.032** |
| Age (year) | 0.991 (0.979 — 1.002) | 0.111 |  |  |  |
| SBP | 0.992 (0.985 — 0.999) | 0.027 |  | 0.995 (0.987 — 1.002) | 0.183 |
| DBP | 1.001 (0.996 — 1.007) | 0.621 |  |  |  |
| T2DM duration |  |  |  |  |  |
| Newly新 | Reference |  |  | Reference |  |
| ＜1 year | 1.677 (0.625 — 4.502) | 0.305 |  | 2.318 (0.758 — 7.091) | 0.141 |
| 1~3 year | 0.382 (0.180 — 0.813) | **0.012** |  | 0.460 (0.205 — 1.036) | 0.061 |
| 3~5 year | 0.646 (0.305 — 1.366) | 0.253 |  | 0.656 (0.297 — 1.449) | 0.298 |
| 5~10 years | 0.589 (0.300 — 1.158) | 0.125 |  | 0.657 (0.318 — 1.354) | 0.255 |
| ＞10 years | 0.487 (0.261 — 0.908) | **0.024** |  | 0.685 (0.343 — 1.368) | 0.283 |
| Drinking |  |  |  |  |  |
| Yes | Reference |  |  | Reference |  |
| No | 0.762 (0.568 — 1.024) | **0.071** |  | 1.031 (0.661 — 1.609) | 0.892 |
| Smoking |  |  |  |  |  |
| Yes | Reference |  |  | Reference |  |
| No | 0.752 (0.550 — 1.028) | **0.074** |  | 0.920 (0.576 — 1.469) | 0.726 |
| Anti-diabetes drugs use |  |  |  |  |  |
| Metformin |  |  |  |  |  |
| Yes | Reference |  |  |  |  |
| No | 0.933 (0.700 — 1.244) | 0.637 |  |  |  |
| Sulfonylurea |  |  |  |  |  |
| Yes | Reference |  |  |  |  |
| No | 1.005 (0.764 — 1.322) | 0.971 |  |  |  |
| Glinides |  |  |  |  |  |
| Yes | Reference |  |  |  |  |
| No | 1.092 (0.497 — 2.400) | 0.826 |  |  |  |
| Thiazolidinediones |  |  |  |  |  |
| Yes | Reference |  |  |  |  |
| No | 0.915 (0.533 — 1.568) | 0.746 |  |  |  |
| α-glucosidase inhibitor |  |  |  |  |  |
| Yes | Reference |  |  | 抑 |  |
| No | 0.771 (0.548 — 1.084) | 0.134 |  |  |  |
| SGLT2 inhibitor |  |  |  |  |  |
| Yes | Reference |  |  | 胰 |  |
| No | 0.899 (0.577 — 1.401) | 0.638 |  |  |  |
| DPP-4 inhibitor |  |  |  |  |  |
| Yes | Reference |  |  |  |  |
| No | 0.906 (0.558 — 1.469) | 0.688 |  |  |  |
| GLP-1 agonist |  |  |  |  |  |
| Yes | Reference |  |  |  |  |
| No | 0.393 (0.116 — 1.334) | 0.134 |  |  |  |
| Insulin |  |  |  |  |  |
| Yes | Reference |  |  | Reference |  |
| No | 1.491 (1.142 — 1.947) | **0.003** |  | 1.228 (0.897 — 1.682) | 0.199 |

**Table S3 Comparison of general indexes between two groups**

|  | T2DM with  Lac＜2.2mmol/L  n=797 | T2DM with  Lac＞=2.2mmol/L  n=797 | *P* value |
| --- | --- | --- | --- |
| BMI（Kg/cm^2^） | 24.2（21.9-26.0） | 24.9（22.7-26.9） | 0.026^*^ |
| VFD (cm^2^) | 70.5（47.8-100.3） | 80.0（55.0-106.0） | 0.012^*^ |
| GLU (mmol/L) | 9.83（7.09-14.82） | 12.88（8.76-18.53） | 0.000^*^ |
| HbA1C（%） | 8.1（6.9-10.8） | 9.5（7.7-11.5） | 0.000^*^ |
| RBC（×10^12^/L） | 4.27（3.8-4.7） | 4.5（4.1-4.9） | 0.000^*^ |
| HGB（g/L） | 129（111-141） | 137（124-149） | 0.000^*^ |
| PLT（×10^9^/L） | 193.5（155.8-230.0） | 201.0（165.0-242.5） | 0.016^*^ |
| TC (mmol/L) | 4.5（3.7-5.4） | 4.4（3.7-5.3） | 0.824 |
| TG (mmol/L) | 1.4（1.0-2.0） | 1.7（1.2-2.7） | 0.000^*^ |
| HDL-c (mmol/L) | 1.2（0.9-1.4） | 1.1（0.9-1.3） | 0.000^*^ |
| LDL-c (mmol/L) | 2.7（2.0-3.5） | 2.6（1.9-3.3） | 0.273 |
| Apo-A1 (mmol/L) | 1.4(1.2-1.6) | 1.3(1.13-1.5) | 0.000^*^ |
| Apo-B (mmol/L) | 0.9(0.7-1.0) | 0.9(0.7-1.01) | 0.465 |
| ALT（U/L） | 19.4（14.7-27.6） | 21.2（15.8-32.0） | 0.002* |
| AST（U/L） | 20.6（16.4-25.4） | 20.3（16.23-27.3） | 0.708 |
| GGT（U/L） | 19.0（14.0-32.75） | 26.0（16.23-27.3） | 0.000^*^ |
| Scr（μmol/L） | 68.1（54.6-88.95） | 64.6（53.2-80.6） | 0.053 |
| UA（μmol/L） | 321.0（258.9-373.0） | 325.4（269.2-397.1） | 0.026^*^ |
| GFR（ml/min） | 93.0（65.3-103.8） | 96.4（80.2-106.9） | 0.001^*^ |

BMI: Body Mass Index; VFD: visceral fat deposition; GLU: Blood Glucose; HbA1c: Glycated Hemoglobin; RBC: Red Blood Cells; HGB: Hemoglobin; PLT: Platelets; TC: Total Cholesterol; TG: Triglycerides; HDL-c: High-density lipoprotein cholesterol; LDL-c: Low-density lipoprotein cholesterol; Apo-A1: Apolipoprotein A1; Apo-B: Apolipoprotein B; ALT: Alanine Aminotransferase; AST: Aspartate Aminotransferase; GGT: Gamma-glutamyltransferase; Scr: Serum creatinine; UA: Uric acid; GFR: Glomerular filtration rate.

**Table S4 Linear regression analysis of metabolic factors affecting lactic acid**

|  | Simple linear regression | |  | Stepwise multiple linear regression | |
| --- | --- | --- | --- | --- | --- |
|  | β | *P*值 |  | β | *P*值 |
| BMI（Kg/cm^2^） | 0.022 | 0.016^*^ |  | - | - |
| VFD (cm^2^) | 0.003 | 0.001^*^ |  | - | - |
| GLU (mmol/L) | 0.020 | 0.000^*^ |  | 0.021 | **0.000^*^** |
| HbA1C（%） | 0.030 | 0.038^*^ |  | - | ^-^ |
| RBC（×10^12^/L） | 0.064 | 0.002^*^ |  | - | - |
| HGB（g/L） | 0.011 | 0.000^*^ |  | 0.063 | 0.166 |
| PLT（×10^9^/L） | - | - |  | - | - |
| TC (mmol/L) | - | - |  | - | - |
| TG (mmol/L) | 0.082 | 0.000^*^ |  | 0.087 | **0.000^*^** |
| HDL-c (mmol/L) | -0.293 | 0.000^*^ |  | -0.015 | 0.748 |
| LDL-c (mmol/L) | - | - |  | - | - |
| Apo-A1 (mmol/L) | -0.287 | 0.006^*^ |  | - | - |
| Apo-B (mmol/L) | 0.155 | 0.036^*^ |  | - | - |
| ALT（U/L） | 0.005 | 0.001^*^ |  | 0.041 | 0.362 |
| AST（U/L） | - | - |  | - | - |
| GGT（U/L） | 0.001 | 0.015^*^ |  | 0.004 | 0.938 |
| Scr（μmol/L） | -0.003 | 0.000^*^ |  | -0.058 | 0.194 |
| UA（μmol/L） | - | - |  | - | - |
| GFR（ml/min） | 0.003 | 0.001^*^ |  | 0.024 | 0.601 |

**Table S5 Comparison of the results of cardiac indicators between two groups**

|  | T2DM with  Lac＜2.2mmol/L  n=797 | T2DM with  Lac＞=2.2mmol/L  n=797 | *P* value |
| --- | --- | --- | --- |
| CK-MB（μg/L） | 1.82（1.31-2.53） | 1.56（1.06-2.25） | **0.000^*^** |
| hs-TNT（μg/L） | 0.01（0.006-0.02） | 0.009（0.006-0.016） | **0.048^*^** |
| NT-proBNP（ng/L） | 110.6（40.2-365.35） | 70.4（31.75-177.8） | **0.000^*^** |
| MYO（μg/L） | 29.17（21-56.51） | 28.52（21-44.14） | **0.023^*^** |
| IVS（mm） | 10.0（9.0-11.5） | 10.0（9.0-11.5） | 0.933 |
| LVDd（mm） | 46（43-48） | 46（43-48） | 0.721 |
| LVDs（mm） | 29.0（27.0-31.0） | 29.0（27.0-31.0） | 0.718 |
| LVPW（mm） | 10.00（9.00-10.00） | 10.00（9.00-10.00） | 0.418 |
| E/A ratio | 0.75（0.65-0.90） | 0.73（0.64-0.85） | **0.034^*^** |

CK-MB: Creatine kinase-MB isoenzyme; hs-TNT: High-sensitivity troponin T; NT-proBNP: N-terminal pro-B-type natriuretic peptide; MYO: Myoglobin; LVDd: Left ventricular end-diastolic volume

**Table S6 Correlation analysis between blood lactic acid level and cardiac indicators**

|  | Simple linear  regression | |  | Stepwise multiple linear regression | |
| --- | --- | --- | --- | --- | --- |
|  | β | *P*值 |  | β | *P*值 |
| CK-MB（μg/L） | -0.170 | 0.000^*^ |  | -0.171 | **0.000^*^** |
| hs-TNT（μg/L） | -0.120 | 0.000^*^ |  | -12.610 | **0.014^*^** |
| NT-proBNP（ng/L） | -0.208 | 0.000^*^ |  | -0.142 | **0.000^*^** |
| MYO（μg/L） | -0.103 | 0.002^*^ |  | -0.097 | **0.004^*^** |
| LVDd（mm） | -0.065 | 0.033^*^ |  | -0.086 | **0.004^*^** |
| E/A ratio | -0.093 | 0.002^*^ |  | -0.070 | **0.021^*^** |

Model 1: unadjusted; Model 2: adjusted based on gender, age, BMI, SBP, and DBP.

**Table S7 Baseline characteristics of all patients in the training set and validation set**

| Characteristics | Training set | Validation set | P value |
| --- | --- | --- | --- |
| n | 599 | 259 |  |
| Sex, n (%) |  |  | 0.774 |
| Male | 329 (54.9%) | 145 (56%) |  |
| Female | 270 (45.1%) | 114 (44%) |  |
| Age (years), median (IQR) | 59 (53, 69) | 61 (52, 69) | 0.951 |
| Drinking, n (%) |  |  | 0.545 |
| Yes | 200 (33.4%) | 81 (31.3%) |  |
| No | 399 (66.6%) | 178 (68.7%) |  |
| Smoking, n (%) |  |  | 0.389 |
| Yes | 434 (72.5%) | 195 (75.3%) |  |
| No | 165 (27.5%) | 64 (24.7%) |  |
| T2DM duration, n (%) |  |  | 0.998 |
| Newly新 | 46 (7.7%) | 21 (8.1%) |  |
| ＜1 year | 37 (6.2%) | 16 (6.2%) |  |
| 1~3 year | 43 (7.2%) | 17 (6.6%) |  |
| 3~5 year | 57 (9.5%) | 23 (8.9%) |  |
| 5~10 years | 114 (19%) | 49 (18.9%) |  |
| ＞10 years | 302 (50.4%) | 133 (51.4%) |  |
| Fructosamine (mmol/L), median (IQR) | 2.69 (2.265, 3.205) | 2.7 (2.315, 3.225) | 0.822 |
| Lactate (mmol/L), median (IQR) | 2.79 (2.23, 3.51) | 2.77 (2.28, 3.345) | 0.675 |
| TG, median (IQR) | 1.7 (1.2, 2.6) | 1.7 (1.2, 2.6) | 0.953 |
| SBP, median (IQR) | 137 (123, 152) | 139 (125, 153) | 0.218 |
| DBP, median (IQR) | 79 (71, 86) | 80 (72, 87) | 0.221 |
| BMI, median (IQR) | 24.8 (22.7, 26.7) | 24.7 (22.6, 26.9) | 0.642 |
| Metformin, n (%) |  |  | 0.006 |
| No | 257 (42.9%) | 85 (32.8%) |  |
| Yes | 342 (57.1%) | 174 (67.2%) |  |
| Sulfonylureas, n (%) |  |  | **< 0.001** |
| No | 426 (71.1%) | 152 (58.7%) |  |
| Yes | 173 (28.9%) | 107 (41.3%) |  |
| Glinides, n (%) |  |  | 0.319 |
| No | 589 (98.3%) | 252 (97.3%) |  |
| Yes | 10 (1.7%) | 7 (2.7%) |  |
| Thiazolidines, n (%) |  |  | 0.925 |
| No | 563 (94%) | 243 (93.8%) |  |
| Yes | 36 (6%) | 16 (6.2%) |  |
| Glycosidase Inhibitors, n (%) |  |  | 0.234 |
| No | 499 (83.3%) | 207 (79.9%) |  |
| Yes | 100 (16.7%) | 52 (20.1%) |  |
| SGLT2 inhibitors, n (%) |  |  | 0.813 |
| No | 551 (92%) | 237 (91.5%) |  |
| Yes | 48 (8%) | 22 (8.5%) |  |
| DPP4 inhibitors, n (%) |  |  | 0.847 |
| No | 555 (92.7%) | 239 (92.3%) |  |
| Yes | 44 (7.3%) | 20 (7.7%) |  |
| Insulin, n (%) |  |  | **< 0.001** |
| No | 406 (67.8%) | 145 (56%) |  |
| Yes | 193 (32.2%) | 114 (44%) |  |

**Table S8: General characteristics of the patients and logistic regression analyses for screening predictors.**

| Characteristics | T2DM with  E/A＜=1  (n=488) | T2DM with  E/A＞1  (n=111) | P  value | Odds Ratio (95% CI) | P value |
| --- | --- | --- | --- | --- | --- |
| Sex, n (%) |  |  | 0.006 |  |  |
| Male | 255 (52.3%) | 74 (66.7%) |  | Reference |  |
| Female | 233 (47.7%) | 37 (33.3%) |  | 1.827 (1.185 - 2.817) | **0.006** |
| Age (years), median (IQR) | 63 (55, 70) | 52 (47, 59) | < 0.001 | 1.090 (1.066 - 1.114) | **< 0.001** |
| Drinking, n (%) |  |  | 0.046 |  |  |
| No | 334 (68.4%) | 65 (58.6%) |  | Reference |  |
| Yes | 154 (31.6%) | 46 (41.4%) |  | 0.652 (0.427 - 0.995) | **0.047** |
| Smoking, n (%) |  |  | 0.202 |  |  |
| No | 359 (73.6%) | 75 (67.6%) |  | Reference |  |
| Yes | 129 (26.4%) | 36 (32.4%) |  | 0.749 (0.480 - 1.169) | 0.203 |
| T2DM duration, n (%) |  |  | 0.039 |  |  |
| Newly | 36 (7.4%) | 10 (9%) |  | Reference |  |
| ＜1 year | 24 (4.9%) | 13 (11.7%) |  | 0.513 (0.194 - 1.357) | 0.178 |
| 1-3 years | 32 (6.6%) | 11 (9.9%) |  | 0.808 (0.303 - 2.153) | 0.670 |
| 3-5 years | 46 (9.4%) | 11 (9.9%) |  | 1.162 (0.444 - 3.037) | 0.760 |
| 5-10 years | 92 (18.9%) | 22 (19.8%) |  | 1.162 (0.501 - 2.693) | 0.727 |
| ＞10 years | 258 (52.9%) | 44 (39.6%) |  | 1.629 (0.754 - 3.518) | 0.214 |
| Fructosamine (mmol/L), median (IQR) | 2.68 (2.2875, 3.1425) | 2.94 (2.185, 3.415) | 0.223 | 0.835 (0.625 - 1.116) | 0.224 |
| Lactate (mmol/L), median (IQR) | 2.865 (2.28, 3.5625) | 2.52 (2.165, 3.085) | 0.002 | 1.571 (1.204 - 2.050) | **< 0.001** |
| TG, median (IQR) | 1.7 (1.2, 2.5) | 1.6 (1.1, 2.75) | 0.802 | 1.012 (0.918 - 1.116) | 0.805 |
| SBP, median (IQR) | 139 (125, 153) | 125 (115.5, 144) | < 0.001 | 1.025 (1.013 - 1.037) | **< 0.001** |
| DBP, median (IQR) | 79.5 (72, 86) | 74 (67, 81) | < 0.001 | 1.032 (1.012 - 1.052) | **0.001** |
| BMI, median (IQR) | 24.9 (22.775, 26.7) | 24.1 (22, 26.7) | 0.176 | 1.042 (0.981 - 1.106) | 0.186 |
| Biguanide, n (%) |  |  | 0.353 |  |  |
| No | 205 (42%) | 52 (46.8%) |  | Reference |  |
| Yes | 283 (58%) | 59 (53.2%) |  | 1.217 (0.804 - 1.840) | 0.353 |
| Sulfonylureas, n (%) |  |  | 0.633 |  |  |
| No | 345 (70.7%) | 81 (73%) |  | Reference |  |
| Yes | 143 (29.3%) | 30 (27%) |  | 1.119 (0.705 - 1.776) | 0.633 |
| Glinides, n (%) |  |  | 0.595 |  |  |
| No | 481 (98.6%) | 108 (97.3%) |  | Reference |  |
| Yes | 7 (1.4%) | 3 (2.7%) |  | 0.524 (0.133 - 2.059) | 0.355 |
| Thiazolidines, n (%) |  |  | 0.557 |  |  |
| No | 460 (94.3%) | 103 (92.8%) |  | Reference |  |
| Yes | 28 (5.7%) | 8 (7.2%) |  | 0.784 (0.347 - 1.769) | 0.557 |
| Glycosidase Inhibitors, n (%) |  |  | 0.066 |  |  |
| No | 400 (82%) | 99 (89.2%) |  | Reference |  |
| Yes | 88 (18%) | 12 (10.8%) |  | 1.815 (0.955 - 3.449) | 0.069 |
| SGLT2 inhibitors, n (%) |  |  | 0.968 |  |  |
| No | 449 (92%) | 102 (91.9%) |  | Reference |  |
| Yes | 39 (8%) | 9 (8.1%) |  | 0.984 (0.462 - 2.097) | 0.968 |
| DPP4 inhibitors, n (%) |  |  | 0.204 |  |  |
| No | 449 (92%) | 106 (95.5%) |  | Reference |  |
| Yes | 39 (8%) | 5 (4.5%) |  | 1.841 (0.709 - 4.784) | 0.210 |
| Insulin, n (%) |  |  | 0.691 |  |  |
| No | 329 (67.4%) | 77 (69.4%) |  | Reference |  |
| Yes | 159 (32.6%) | 34 (30.6%) |  | 1.094 (0.701 - 1.709) | 0.691 |
